# Living at the edge: biogeographic patterns of habitat segregation conform to speciation by niche expansion in *Anopheles gambiae*

**DOI:** 10.1186/1472-6785-9-16

**Published:** 2009-05-21

**Authors:** Carlo Costantini, Diego Ayala, Wamdaogo M Guelbeogo, Marco Pombi, Corentin Y Some, Imael HN Bassole, Kenji Ose, Jean-Marie Fotsing, N'Falé Sagnon, Didier Fontenille, Nora J Besansky, Frédéric Simard

**Affiliations:** 1 Institut de Recherche pour le Développement (IRD), UR016, and Institut de Recherche en Sciences de la Santé (IRSS), 01 BP 171, Bobo-Dioulasso, Burkina Faso; 2 Laboratoire de Lutte contre les Insectes Nuisibles (LIN), Institut de Recherche pour le Développement (IRD), UR016, 911 Av Agropolis, 34394 Cedex 5, Montpellier, France; 3 Centre National de Recherche et de Formation sur le Paludisme (CNRFP), 01 BP 2208, Ouagadougou, Burkina Faso; 4 Parasitology Unit, Dept. Public Health, University of Rome "La Sapienza", P le Aldo Moro 5, 00185, Rome, Italy; 5 Institut International d'Ingénierie de l'Eau et de l'Environnement (2IE), 01 BP 594, Ouagadougou, Burkina Faso; 6 Université de Ouagadougou, 03 BP 7021, Ouagadougou 03, Burkina Faso; 7 Institut de Recherche pour le Développement (IRD), US140, Pôle Géomatique ESPACE-IRD, 5 rue du Carbone, 45072 Cedex 2, Orléans, France; 8 Eck Institute for Global Health, Department of Biological Sciences, 317 Galvin Life Sciences Bldg., University of Notre Dame, Notre Dame, IN 46556-0369, USA; 9 Institut de Recherche pour le Développement (IRD), UR016, and Organisation de Coordination pour la lutte contre les Endémies en Afrique Centrale (OCEAC), B.P. 288, Yaoundé, Cameroon; 10 Organisation de Coordination pour la lutte contre les Endémies en Afrique Centrale (OCEAC), BP 288, Yaoundé, Cameroon

## Abstract

**Background:**

Ongoing lineage splitting within the African malaria mosquito *Anopheles gambiae *is compatible with ecological speciation, the evolution of reproductive isolation by divergent natural selection acting on two populations exploiting alternative resources. Divergence between two molecular forms (M and S) identified by fixed differences in rDNA, and characterized by marked, although incomplete, reproductive isolation is occurring in West and Central Africa. To elucidate the role that ecology and geography play in speciation, we carried out a countrywide analysis of *An. gambiae *M and S habitat requirements, and that of their chromosomal variants, across Burkina Faso.

**Results:**

Maps of relative abundance by geostatistical interpolators produced a distinct pattern of distribution: the M-form dominated in the northernmost arid zones, the S-form in the more humid southern regions. Maps of habitat suitability, quantified by Ecological Niche Factor Analysis based on 15 eco-geographical variables revealed less contrast among forms. M was peculiar as it occurred proportionally more in habitat of marginal quality. Measures of ecological niche breadth and overlap confirmed the mismatch between the fundamental and realized patterns of habitat occupation: forms segregated more than expected from the extent of divergence of their environmental envelope – a signature of niche expansion. Classification of chromosomal arm 2R karyotypes by multilocus genetic clustering identified two clusters loosely corresponding to molecular forms, with 'mismatches' representing admixed individuals due to shared ancestral polymorphism and/or residual hybridization. In multivariate ordination space, these karyotypes plotted in habitat of more marginal quality compared to non-admixed, 'typical', karyotypes. The distribution of 'typical' karyotypes along the main eco-climatic gradient followed a consistent pattern within and between forms, indicating an adaptive role of inversions at this geographical scale.

**Conclusion:**

Ecological segregation between M and S is consistent with niche expansion into marginal habitats by chromosomal inversion variants during early lineage divergence; presumably, this process is promoted by inter-karyotype competition in the higher-quality core habitat. We propose that the appearance of favourable allelic combinations in other regions of suppressed recombination (e.g. pericentromeric portions defining speciation islands in *An. gambiae*) fosters development of reproductive isolation to protect linkage between separate chromosomal regions.

## Background

Ecological diversification is responsible for the adaptive radiation of several groups of organisms that are not separated geographically but segregate in alternative habitats or ecological niches [1,2]. The evolutionary process by which ecological diversification drives species formation, known as ecological speciation, involves the establishment of barriers to gene flow through ecologically-based divergent selection [3,4]. Interbreeding between populations that have ecologically diverged, by either niche specialization or invasion of a new niche, produces hybrid individuals of lower fitness in each of the parental habitats. Ecological speciation theory predicts that reproductive isolation is environment-dependent, i.e. it is driven by ecological selective forces such as resource competition or predation, instead of genetic mechanisms producing hybrid sterility or inviability. The strength of reproductive isolation is correlated with the degree of ecological divergence, rather than time since lineage splitting. Convincing evidence exists for the role of ecological speciation in nature, across a disparate range of taxa from angiosperms to insects and vertebrates [4-7].

Past and ongoing radiation of some members of the *Anopheles gambiae sensu lato *(*s.l*.) complex is considered to reflect different stages of the ecological speciation process, in particular with respect to divergence of the larval aquatic habitat [8]. This complex of African mosquitoes comprises seven recognized isomorphic species distinguished in most cases by fixed chromosomal paracentric inversions, and often by different larval breeding habitats [9,10]. The significance of the complex for evolutionary biologists is compounded by its tremendous importance for human health: this complex contains two of the most important Afrotropical vectors of malaria, a pathology that kills about two million children below the age of five each year in sub-Saharan Africa alone [11]. The two main culprits of this extremely efficient vectorial system are the nominal species *An. gambiae sensu stricto *(*s.s*.), and its sibling *An. arabiensis*, which occur in sympatry across much of their geographical distribution [12]. Reproductive isolation between *An. gambiae s.s*. and *An. arabiensis *is expressed both at the pre-zygotic and post-zygotic level. Laboratory colonies of the two species can be induced to mate and produce viable hybrid progeny, but males have atrophied testes, hence only females are fertile [13]. In nature, adult hybrids are found at an average frequency of 0.02–0.76% [8], and heterogamic matings are rare (our own unpublished data). The occurrence of viable and fertile female hybrids can lead to introgression of genetic material between the two species [14,15]. Despite this semi-permeable nature of the species boundary [16], *An. gambiae s.s*. and *An. arabiensis *maintain many distinct genetic and bionomical features and are taxonomically considered good species under most species concepts.

Ongoing lineage splitting within the nominal species *An. gambiae s.s*. (hereafter, *An. gambiae*) has led to the recognition of "molecular forms" M and S [17,18], which share many characteristics compatible with ecological speciation. Despite the lack of reproductive isolation in captive laboratory strains [19], a significant pre-mating barrier (only 1% of natural inseminations are heterogamic) prevents extensive hybridization in natural populations [20]. The rarity of interbreeding can be explained at least in part by the occurrence of mostly homogamous mating swarms [21]. Hybrids are fully fertile and viable [22], and although rarely found in natural populations [23] (and our own data, see Results), they can account for the high estimates of gene flow and the dearth of significant genetic differentiation between forms inferred from genome-wide scans of natural populations [24,25]. However, two small unlinked centromere-proximal regions of the genome show high levels of divergence across West and Central Africa where M and S populations occur in sympatry [26-29]. The centromere-proximal location protects both regions from recombination in the face of gene flow between M and S, but the patterns of sequence variation are not consistent with reduced recombination acting alone [26,28]. Divergent natural selection aided by reduced recombination in these regions is what nominates them as "speciation islands" that are expected to harbour the genes responsible for emerging ecological and reproductive divergence [28].

Despite compelling evidence for the existence of significant if incomplete reproductive isolation between the M and S forms, the mechanistic basis for their reproductive isolation remains completely unknown at both genetic and ecological levels. Moreover, while the application of high throughput genomic technologies has led to relatively rapid progress in locating regions of major genetic distinction between M and S forms, comparable amounts of ecological evidence concerning the sources of divergent selection have not been forthcoming. In the arid savannas of Mali and Burkina Faso in West Africa, evidence collected prior to the recognition of the M and S molecular forms supported some degree of spatial and temporal segregation between taxonomic units known as "chromosomal forms" [30-32]. The chromosomal forms, defined based on configurations of shared polymorphic chromosomal inversions, correspond imperfectly or not at all with the *An. gambiae *molecular forms [17,33], now considered to be the relevant taxonomic units. Accordingly, much of the bionomics of *An. gambiae *ecotypes needs to be revisited in the context of molecular forms. In much of West and Central Africa, the M and S forms occur in the same villages, sharing the same resources such as hosts, adult resting sites, and freshwater larval breeding sites. At a microgeographic scale, the aquatic larvae of *An. gambiae *and *An. arabiensis*, and of the two molecular forms of *An. gambiae*, have been found at times to segregate in different breeding sites [23,34,35], though the ecological factors modulating larval niche partitioning remain unclear [36]. Habitat segregation of larvae of the two molecular forms has sometimes been associated with the nature of the breeding site. In Burkina Faso, larvae of the M form are more prevalent in larger anthropogenic longer-lasting habitats such as artificial lakes and rice fields, whereas the S form predominates in rain-dependent smaller ephemeral habitats such as puddles and road ruts [37,38]. Habitat-dependent fitness traits have been attributed to larval predation and inter-form competition [39,40].

Spatial scale is an important ecological factor structuring communities and populations [e.g. [41,42]]. Because geography has an impact not only on the likelihood of gene flow but also on the ecological sources of divergent selection [4], habitat segregation between M and S should be studied also at larger spatial scales. Across the African continent, knowledge of the geographical distribution of the molecular forms is sparse. della Torre and colleagues reviewed existing data on the occurrence and chromosomal make-up of molecular forms across Africa and its offshore islands, and showed that both are present in all major biomes and geographic areas, except for the absence of M east of the Rift Valley [33] (although there is a single record of the presence of the M form east of the Rift Valley based on a few individuals collected in Zimbabwe [43]). However, a higher-resolution, systematic analysis of the distribution and relative abundance of M and S over large spatial ranges is still lacking: are the two molecular forms equally abundant across major eco-climatic regions, or do they segregate at this eco-geographical scale? How does the present geographical distribution relate to the ecological factors driving the speciation process? Can we expect hybridization and gene flow between M and S to be frequency-dependent across their range, in accordance with their geographic distribution?

According to the general tenets of ecological speciation, several predictions can be put forward concerning the ecological divergence of the siblings and incipient species of the *An. gambiae *complex. First, it can be predicted that in the course of their radiation all the three taxa should have diverged ecologically to some extent. Second, from the positive association between the strength of ecological divergence and reproductive isolation found in other taxa, it is expected that the ecological niche of the two molecular forms of *An. gambiae *should have diverged less than that of *An. gambiae *and *An. arabiensis*. Third, since the molecular forms of *An. gambiae *represent a more recent realization of an ongoing speciation process, with the M-form likely being most recently derived [8,10], we predict that the M-form should occur in more marginal habitats than the other two taxa, in accordance with a process of niche expansion from the original, optimal habitat [[44], for an example, see [45]].

In this and the companion paper by Simard and colleagues [46], we present the results of two countrywide surveys of *An. gambiae *M, S and *An. arabiensis *carried out in Burkina Faso (West Africa) and Cameroon (Central Africa) designed to test these predictions with a biogeographic approach [47] based on ecological niche modelling [48,49]. The patterns emerging from these studies help elucidate the role that ecology and geography play in ongoing speciation between the molecular forms of *An. gambiae*. Specifically, we asked the following five questions: (i) do the three cryptic taxa segregate ecologically at a countrywide spatial range, and to what extent does their habitat overlap? (ii) what eco-geographical predictors characterize the fundamental ecological niche of the three taxa, and which key ecological factors discriminate among them? (iii) does the extent of habitat segregation correlate positively with the degree of reproductive isolation? (iv) does the M form occur in more marginal habitats than the S form? (v) do chromosomal inversions play a role in the ecological segregation of the molecular forms of *An. gambiae*? To answer these questions, we implemented an empirical approach to distribution modelling using two spatially explicit statistical methods. First, to identify the ecological forces at work in the speciation process, we performed an Ecological Niche Factor Analysis [50] to infer both the eco-geographical factors delimiting the fundamental ecological niche of a focal species and the potential habitat suitability across the entire study area. Second, to explore the extent of realized geographical segregation and habitat overlap, we employed geostatistical methods to construct kriged surfaces of the relative abundance of the three cryptic taxa. By comparing the extent of realized versus potential occurrence and abundance of the three taxa in relation to predicted habitat quality, we tested whether the distribution of M (the most recent taxon) is consistent with a process of niche expansion into a habitat of marginal quality. Finally, we compared the degree of association between chromosomal variants and environmental predictors by multivariate ordination techniques to characterize the ecological niche of the different karyotypes observed in our study area, and verify whether chromosomal inversions play a role in the ecological segregation of the molecular taxa of *An. gambiae*.

The parallel approach in Burkina Faso and Cameroon was prompted by the dramatic distinctions between these countries, both in the extent of eco-geographical diversity and the level of chromosomal inversion polymorphism observed in the molecular taxa: Cameroon covers a wide range of biomes including the equatorial rainforest, and the mesic to xeric savannas of Central Africa, while Burkina Faso lies in the arid Sudanese savanna belt of West Africa. Chromosomally, the forest populations are largely monomorphic standard, whereas savanna populations are highly polymorphic [18,51-54]. We expected that this comparative approach would be revealing about the forces driving ecological speciation, in light of the long-hypothesized relationship between chromosomal inversions, ecological adaptation, and speciation in *An. gambiae*. In this paper we present the results of the Burkina Faso survey; those of the Cameroon survey are presented in the companion paper [46].

## Methods

### Study area and sampling plan

**Figure 1:**
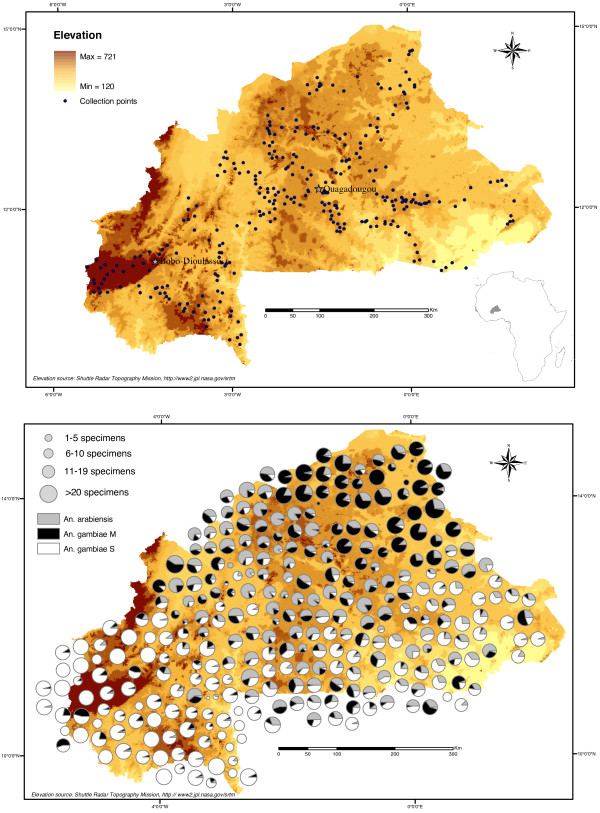
**Study area and observed relative abundance of members of the *Anopheles gambiae *complex in Burkina Faso**. Map of sampled locations (above), with pies showing results of molecular identifications (below) expressed as relative frequencies of members of the *An. gambiae s.l*. complex (shading inside the pie), and total sample size (size of the pie) from each location.

A countrywide survey in Burkina Faso was performed across an area spanning LAT 9°45'N–14°40'N and LONG 5°30'W–1°45'E (Figure 1). In Burkina Faso, annual rainfall and the duration of the rainy season vary following a latitudinal gradient: in the northernmost regions, rainfall does not exceed 300 mm during 2–3 months; in the southernmost regions rainfall can be as high as 1200 mm during 4–5 months. Population size and relative frequencies of *An. arabiensis *and molecular forms of *An. gambiae *vary seasonally according to the distribution of rains [38]. To maximise the chance of sampling all three taxa from each location across the country, the mosquito survey began in the north at the peak of the rainy season (2 August) and continued until the end of the rainy season in the south (26 October).

A stratified random sampling scheme was adopted with the objective of selecting sites (villages) that were representative of the eco-geographical diversity of Burkina Faso and also accessible during the rainy season. This was achieved by defining three transects covering the full diversity of eco-climatic regions and landscapes present in the country, following the major network of roads. In total, three hundred villages were chosen for sampling (additional file 1) from the national topographic database (*Banque Nationale des Données Topographiques *– BNDT) of the Institute of Geography of Burkina Faso (*Institut Géographique du Burkina *– IGB), an electronic georeferenced database of all populated places published in 2005 by IGB. These villages were chosen at random, based on a grid of 5 × 5 km cells superimposed on other topographical layers in a geographical information system (GIS). For each transect, a buffer zone of 15 km on each side was selected and a number of cells proportional to the length of each transect was randomly chosen. One village per cell was then selected at random from the list of villages available within the chosen cells.

### Entomological survey

Freshly-fed female *An. gambiae s.l*. were collected in the morning while resting inside human dwellings by manual aspiration with the aid of electrical aspirators. Mosquitoes were kept in small cages wrapped in wet towels and stored inside cool boxes. Additionally, indoor insecticide space-sprays were carried out in the early afternoon. Collected mosquitoes were morphologically identified in the field under a dissecting microscope. Ovaries of *An. gambiae s.l*. females at the appropriate stage for polytene chromosome analysis were immediately cropped and placed in a fixative agent (Carnoy solution, 1:3 glacial acetic acid:absolute ethanol) contained within individual 1.5-mL reaction microtubes labelled with a unique serial number. The remaining carcass was placed in an identically numbered individual 1.5-mL microtube containing a desiccant (silica gel). Ovaries were stored at -20°C, and carcasses maintained at ambient temperature before further processing. Mosquito DNA was extracted from the carcass and identified to species and molecular form using rDNA-based PCR assays [55,56]. The corresponding ovaries were prepared for karyotype analysis according to standard procedures [57]. The banding pattern was observed under a phase-contrast microscope (400×) and interpreted with reference to the chromosomal map and nomenclature of Coluzzi and colleagues [9,10].

### Geostatistical analysis

Geostatistical interpolators were used to construct surfaces of the distribution and relative abundance of members of the *An. gambiae s.l*. complex at unsampled locations. Two response variables were modelled: (i) the relative frequency of *An. arabiensis vs. An. gambiae s.s*. and (ii) the relative frequency of molecular form M *vs*. S. Spatial modelling was performed in the *Geostatistics *module of ArcGIS^® ^v. 8.3 (ESRI, 2002; ) by fitting an anisotropic spherical function of the arcsine-transformed response variable to the semivariogram. Interpolation was then carried out by universal kriging.

### Association analysis

To investigate patterns of co-occurrence among taxa at the countrywide level, we calculated the degree of similarity in the occurrence of species pairs across locations by the *V *association coefficient [58]. This index is based on the frequency of joint presences and absences by two species in a two-by-two contingency table. The coefficient ranges from -1 to +1, the sign of the coefficient denoting whether the species co-occur more (positive sign) or less (negative sign) than expected at random (*V *= 0) under given species frequencies. The index is called the point correlation coefficient, because it is equal to the Pearson correlation coefficient between two species when the values of one and zero are used to denote their presence or absence, respectively. To assess the statistical significance of the index (null hypothesis: *V *= 0), we calculated 95% confidence intervals by bootstrapping the *V *values by location over 5,000 replicates.

### Ecological Niche Factor Analysis (ENFA)

Static species distribution models (SDM) make predictions about the probability of occurrence and geographical distribution of a focal species by extrapolating to a larger spatial extent the association between the environment and the focal species found at a certain number of sample locations [48]. The ENFA is a multivariate SDM technique based on the concept of ecological niche as a *n*-dimensional hypervolume in a hyperspace of *n *resource axes [50,59]. The ENFA extracts *n *uncorrelated factors, constructed as linear combinations of the *n *resource axes, explaining the major part of the environmental distribution of a focal species. The first factor is the *marginality*, describing how far is the optimum for the focal species from the mean environmental profile of a reference set (in our case, the whole of Burkina Faso). A global marginality value ≥1 means that the species lives in a particular habitat relative to the distribution of habitats available in the reference set. The second and subsequent factors represent the *specialization *(*S*) or *tolerance *(1/*S*) factors. They are sorted in decreasing amounts of explained variance, and describe how specialized is the focal species with respect to the range of environments available in the study area. A randomly chosen set of cells is expected to have a global specialization value of one, with values >1 indicating some form of specialization. A univariate interpretation of the ENFA, and a detailed description of its principles and operation are given in [50]. The strength of the ENFA is that it uses only the presence data of a focal species. In distinction to alternative SDM techniques, absence from a location is considered uninformative rather than indicative of habitat unsuitability.

Our study area was rasterized in spatial units of 1-km isometric cells. The entomological data and the eco-geographical variables characterizing the environment were related to each spatial unit and entered into a GIS database in ArcGIS^® ^v. 8.3. After performing the ENFA, a Habitat Suitability Index (HSI), ranging continuously from zero to one, was calculated for each cell with the software *Biomapper *v.4.0 [60]. Then, following Hirzel and colleagues [61], habitat suitability (HS) maps were plotted using four classes of habitat suitability that were defined from the predicted-to-expected (*P*/*E*) ratio of habitat suitability based on the continuous Boyce index evaluator statistic [62]: (1) *unsuitable *habitat, where no presence of the focal species is predicted (*P*/*E *= 0, allowing for the upper 95% confidence limit); (2) *marginal *habitat, where presence is predicted at a frequency less than expected by chance alone (*P*/*E *≤ 1); (3) *suitable *habitat, where presence is predicted at a frequency higher than expected by chance alone; and (4) *optimal *habitat, separated from suitable habitat by the steep rate of change of the *P/E *ratio at the higher end of the spectrum of habitat suitability.

### Eco-geographical predictors

We selected fifteen eco-geographical variables (EGV), belonging to three classes: climate (4 variables), topography (6 variables), and land use (5 variables) (Table 1). The climatic variables were retrieved from the meteorological database of the network of weather stations of Burkina Faso managed by the Agency for the Safety of Aerial Navigation in Africa and Madagascar (ASECNA). Topographic and land use variables were extracted from the BNDT. The quantitative climatic variables and three topographic variables (altitude, aspect, and slope) were post-processed to extrapolate the mean value of each spatial unit cell by interpolation and rasterization. Most EGVs were normalized according to the Box-Cox algorithm prior to analysis. The Boolean variables classifying the occupancy of soil of individual cells by categories of land use were post-processed to render them quantitative: a buffer zone of 5 km radius around the focal cell was drawn and the relative frequency of 1 × 1 km cells within the buffer zone belonging to the category of land use under consideration was calculated. The frequency was then normalized by the arcsine transformation prior to analysis. The size of the buffer zone was guided by the average dispersal distance that can be covered by *An. gambiae *complex mosquitoes in our study area [63]. Quantitative information was extracted from the three vectorial geographical features (populated places: points; transportation network: polylines; and hydrologic network: polygons), by calculating the minimum distance from the focal cell to each of these features.

**Table 1:** Eco-geographical variables selected for the ENFA

**No**.	**Class**	**Code**	**Description**	**Source**
**1**	Climate	RAIN	Mean annual rainfall	ASECNA
**2**	Climate	SUN	Mean annual solar radiation	ASECNA
**3**	Climate	EVAPO	Mean annual evapotranspiration	ASECNA
**4**	Climate	TEMP	Mean annual temperature	ASECNA
**5**	Land use	OPEN	Frequency of cells without tree cover	BNDT
**6**	Land use	CROP	Frequency of cells with annual crops	BNDT
**7**	Land use	FARM	Frequency of cells with farmland other than crops	BNDT
**8**	Land use	SHRUB	Frequency of cells with shrub cover	BNDT
**9**	Land use	FOREST	Frequency of cells with forested areas	BNDT
**10**	Topography	POPPL	Minimum distance from populated places	BNDT
**11**	Topography	ROAD	Minimum distance from roads	BNDT
**12**	Topography	HYDRO	Minimum distance from hydrological features	BNDT
**13**	Topography	ALT	Mean altitude	BNDT
**14**	Topography	SLOPE	Mean slope	BNDT
**15**	Topography	ASPECT	Mean aspect	BNDT

Characteristics of the environmental descriptors used in the Ecological Niche Factor Analysis.

### Evaluation of Habitat Suitability models

Accuracy and robustness of the habitat suitability models was assessed using a 10-fold cross-validation procedure implemented in *Biomapper*. We used the mean and standard deviation as measures of, respectively, central tendency (assessing model accuracy) and dispersion (assessing model robustness) of evaluator statistics. Evaluator statistics of presence-only SDMs like the ENFA are the absolute validation index (*AVI*), the contrast validation index (*CVI*), and the continuous Boyce index [61]. The *AVI *is calculated as the proportion of presence points falling in cells having a threshold habitat suitability index (in our case, HSI = 0.5). The *CVI *is the *AVI *minus the *AVI *of a random model, i.e. one that predicts presence everywhere. Both statistics suffer from the choice of an arbitrary threshold. To overcome this limitation, the continuous Boyce index *B*_cont(20) _is calculated as the Spearman correlation coefficient between the ratio of the predicted over expected frequency of evaluation points, iterated across the range of HSI values over a sliding window of 20 HSI units, and the habitat suitability index. Further details about the calculation, use, and properties of the Boyce index, are given in [61,62] and the companion paper of Simard *et al*. [46].

### Niche breadth and overlap

To compare patterns of similarity, breadth, and overlap of the fundamental and realized ecological niches of each taxon, we adopted two approaches. First, a multivariate discriminant analysis was performed to isolate the environmental ordination axis along which each pair of forms/species was maximally differentiated. Statistics of niche overlap and related measures (derived from [64]) were then calculated on this factor, as in [65]. This analysis was performed in *Biomapper *v.4.0. In addition, the same statistics were calculated from the dataset of location samples.

### Chromosomal polymorphism and population structure

We implemented a Bayesian multilocus genetic clustering approach using the software STRUCTURE v.2.2 [66-68]. STRUCTURE calculates an estimate of Ln [Pr(*X*|*K*)], representing the probability of obtaining the observed genetic data *X *conditional on the presence of *K *populations (i.e. "clusters"), with *K *unknown *a priori*. We adopted the "admixture" model, as its assumption of correlated allele frequencies between populations conformed best to our biological system, given residual gene flow between molecular forms and linkage between some chromosomal inversions. A series of five independent runs were performed for each *K *from 1 to 10, setting 40,000 and 500,000 as burn-in and run numbers (measures of the length of the MCMC algorithm). The probability of each individual to belong to one of the *K *populations was plotted for the most likely value of *K*. The analyses were at first performed without prior assignment of individuals to molecular or chromosomal form to verify the degree of consistency in the classification of karyotypes by the three different approaches/markers. The analyses were then repeated with prior assignment of karyotypes to molecular form to assess the probability of admixture of each individual.

For the purpose of analysis, inverted and standard arrangements were treated as alternative alleles of a bi-allelic locus. However, because the 2R*d *and 2R*u *inversions overlap, we considered that they constituted a three-allelic locus. Moreover, some inversions have closely linked breakpoints producing extreme cases of linkage disequilibrium; thus, we repeated the analysis by grouping inversions in four 'inversion systems': 2R*j*/+, 2R*b*/*bc*/+, 2R*d*/*u*/+ and 2L*a*/+. Results of the two analytical approaches were concordant; here we present only those of the former approach.

### Ecological niche of chromosomal variants

The association between inversion polymorphism and environmental variables in each molecular form at each sampled location was investigated by multivariate ordination with the software CANOCO v. 4.5 [69]. The karyotypes recorded at each sampled location were plotted in ordination space by detrended correspondence analysis (DCA), with environmental variables passively plotted to interpret the general relationship between karyotypes and EGVs. This analytical procedure does not take into account the geographical structure of the data, hence results depend upon the underlying spatial and temporal distribution of the EGVs.

To extract eco-geographical predictors to be used in the DCA, raster maps of the transformed EGVs used for the ENFA were translated into vector format and overlaid on a layer containing the sampled locations in ArcGIS v.8.3. The value of each EGV at every sampled location was extracted and saved in a separate layer. All layers were subsequently collapsed in a single table containing information relative to all the EGVs for each sampled location. Several additional EGVs extracted from the BNDT were included in this analysis: (i) the distance from major water bodies, including large artificial or natural water reservoirs (lakes), areas of extensive rice cultivation, and areas amenable to flooding; (ii) the vegetation zone as defined by landscape-level physiognomic and floristic associations (see additional file 2). The four main zones present in Burkina Faso, i.e. northern Sahelian, southern Sahelian, northern Sudanese, and southern Sudanese, typically characterize a cline from more to less xeric habitats, respectively; (iii) the habitat suitability for either M or S classified as Optimal, Suitable, Marginal, or Unsuitable (see above); (iv) the relative frequency of 'competitors' for a focal taxon (i.e. the other *An. gambiae s.l*. taxa).

In the tabular input for CANOCO analyses, "samples" were the individual locations, and "species" were the individual karyotypes recorded on the 2R chromosomal arm of each molecular form. The karyotype at chromosomal arm 2R of each individual was scored as a series of five digits, after [31]. Each digit represented one of five polymorphic inversions common in our study area (2R*j*-2R*b*-2R*c*-2R*d*-2R*u*), and could assume a value of "0", "1" or "2". The value "0" indicated the standard homokaryotype, "1" the heterokaryotype, and "2" the inverted homokaryotype with respect to each inversion. Inversion 2L*a *on the left arm of chromosome 2 was not considered in the analyses because it was nearly fixed (frequency ≥0.95) across the study area. Due to the limited number of specimens collected in each sampled location, the "species" table contained many zero values. To avoid biases due to the sparseness of the table, we implemented a square root transformation and downweighted rare 'species' (i.e. karyotypes) by assigning a weight to each karyotype prior to analysis.

## Results

### Mosquito identification

Collections in most villages yielded our target of twenty dissected female *An. gambiae s.l*.(inter-quartile range 16–23; cf. additional file 1). Overall, 4,896 specimens were molecularly identified out of 5,056 that were processed. Of the identified specimens, most were *An. gambiae *molecular form S (49.2%), followed by the M form (28.9%) and *An. arabiensis *(20.9%). We found one *An. gambiae-An. arabiensis *hybrid, and 46 M-S hybrids, representing 0.02% and 0.94%, respectively, of all identified specimens. Unless indicated otherwise, the hybrids were omitted from further analyses.

### Geographical distribution

The relative frequency of the three taxa across the sampled locations is presented in Figure 1. The distribution of M and S followed a clear geographical pattern: the M form dominated in the northernmost arid zones; the S form was generally most abundant in the moister southern regions. *An. arabiensis *was more prevalent in the northwest and in the central plateau – between 11°30'N and 13°30'N – decreasing in abundance when moving away from this area.

**Figure 2:**
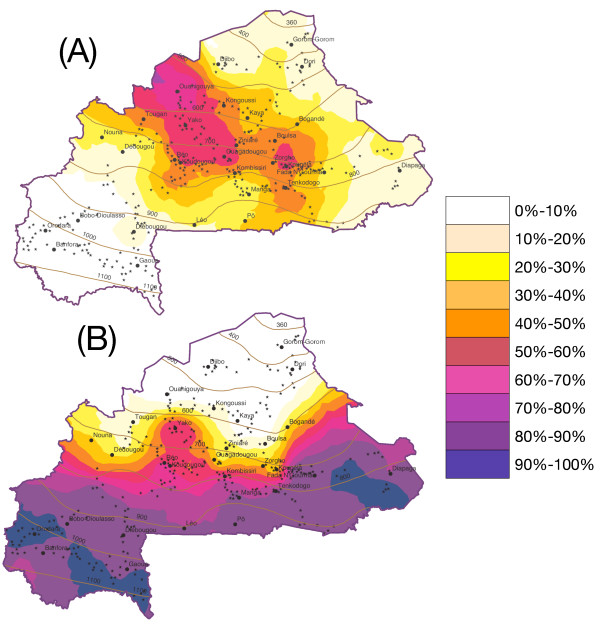
**Interpolated relative abundance of members of the *Anopheles gambiae *complex in Burkina Faso**. Maps of the kriged relative frequency of members of the *Anopheles gambiae *complex across Burkina Faso: A) *An. arabiensis *(*vs. An. gambiae*); B) *An. gambiae *molecular form S (*vs*. form M). The figure also shows major populated places (>10,000 inhabitants; labelled dots), and sampled locations used as interpolators (stars). Continuous lines denote mean annual rainfall isohyets for the period 1970–2000.

The interpolated relative frequencies of *An. arabiensis vs. An. gambiae *(both forms combined) and M *vs*. S form (Figure 2) quantified the patterns observed in Figure 1. In contrast to expectation based on its continental distribution, *An. arabiensis *was not most abundant in the arid sahelian savanna in the north. Rather, this species did not predominate in any region, except perhaps for a small patch (3% of the total study area) in the north-west around the town of Ouahigouya (Figure 2A). Perhaps even more interestingly, this species remained at intermediate levels of relative abundance across much of the central and southern region occupied largely by the catch basin of the river Nakambé (formerly the White Volta). This is the most densely populated area in Burkina Faso, where anthropogenic modifications and population pressure on the natural environment are at their highest.

The two molecular forms were distributed according to opposite latitudinal clines (Figure 2B), with M dominating (relative frequency ≥70%) in the north (representing 29% of the total surface), and S in the south (50% of the total surface). The reversal in frequency between the two forms was rather abrupt between 12°N and 13°N latitude (21% of the total surface), with the exception of an area of approx. 50 km radius between the towns of Koudougou (12°15'43"N 2°22'24"W) and Yako (12°57'33"N 2°16'03"W). Although the two forms actually co-existed in sympatry across most, if not all, of the study area, the rather steep transition of relative abundance was not matched by equally steep changes of environmental features across the 'boundary' (maps of EGVs not shown), suggesting that this distribution pattern may have resulted not only from the underlying response of the two molecular forms to the environment, but also from, at least to some degree, spatial dependence and aggregative dynamics due to biological processes such as competitive interactions between forms, predation, or dispersal – among others [70].

### Association analysis

The similarity of distribution of the three taxa among the samples was investigated by the point correlation coefficient *V*, which measures the degree of species association based on presence/absence. It is generally assumed that similarity in species occurrence among samples reflects similarity in their overall ecological behaviour [58]. However, as measures of species association depend upon the chosen set of samples [58], we performed three distinct analyses to explore the role of geographical scale and ecological conditions on the degree of association and similarity. First, we used the whole data set of samples from the entire study area to investigate the macroecological pattern of species occurrence that was examined previously biogeographically using geostatistical interpolators. Then, because of the gross latitudinal pattern in species distribution (Figures 1 and 2), we stratified the analysis in a northern (LAT>12°30'N), and a southern (LAT<12°30'N) stratum, whose boundary corresponds approximately to the reversal in molecular forms relative abundance (Figure 2).

The analysis of the whole data set confirmed the pattern that could be inferred from the maps of species distribution and abundance in Figure 2: *Anopheles arabiensis *and *An. gambiae *M were positively associated, whereas both these taxa were negatively associated with *An. gambiae *S ("Total" row in Table 2), indicating that the two molecular forms of *An. gambiae *were overall ecologically more dissimilar than the M form and *An. arabiensis*. The southern stratum association analysis conformed exactly to the same pattern (Table 2). Conversely, in the northern stratum the 95% bootstrap confidence intervals of the point correlation coefficient overlapped *V *= 0 in two cases out of three, suggesting that in this area *An. gambiae *S occurred independently of the other two taxa (Table 2). A weak, and possibly statistically significant, negative association between *An. gambiae *M and *An. arabiensis *was detected in this region (Table 2). The fact that *An. gambiae *M and *An. arabiensis *co-occurred less than expected under a null model where they were most abundant, and were associated more than expected where they were least abundant suggests that they have more similar ecologies (e.g. adaptation to more xeric conditions), so that they occur in the same habitats where they presumably engage in frequency-dependent competitive interactions. Conversely, the fact that *An. gambiae *S was significantly less associated than expected where it was the most abundant, and it was distributed independently of the other taxa where it was the least abundant, suggests that it is the strongest competitor of the three taxa.

**Table 2:** Association analysis of species co-occurrence

**Latitude**	***N***	*An. arabiensis vs. An. gambiae *S	*An. arabiensis vs. An. gambiae *M	*An. gambiae *M *vs. An. gambiae *S
**>12°30'N**	102	+0.13 (-0.07, +0.31)	-0.06 (-0.09, -0.03)*	-0.06 (-0.20, +0.16)*
**<12°30'N**	203	-0.13 (-0.18, -0.08)	+0.25 (+0.10, +0.39)	-0.15 (-0.20, -0.09)
**Total**	305	-0.11 (-0.18, -0.02)	+0.27 (+0.14, +0.39)	-0.22 (-0.27, -0.16)

* confidence limits are approximate because some Monte Carlo replications returned indeterminate values of the *V *coefficient due to the occurrence in the 2 × 2 contingency table of more than one cell with zero values.

Association coefficients *V *(95% bootstrap confidence limits) assessing the degree of co-occurrence of species pairs across sampled locations, stratified by two latitudinal classes representing regions of contrasting levels of relative abundance of the two molecular forms of *An. gambiae s.s*. (cf. Figures 1 and 2). *N* = number of sampled locations (i.e. villages).

### Ecological Niche Factor Analysis

Sample size is the most critical parameter when choosing a sampling strategy optimized to return accurate predictions by the ENFA [71]. Our analysis was based on a variable number of presence data points out of 300 sampled locations depending on each taxon: 238 for *An. arabiensis*, 234 for the M form, and 251 for the S form; in all cases our sample size approximated values returning robust results in simulations using virtual scenarios [71-73]. The global marginality, specialisation, and tolerance indices did not differ among the three taxa, but the indices indicated that their ecological requirements were rather marginal and specialized with respect to the global distribution of the EGV values in our reference set, i.e. that defined by the environmental conditions encountered across the whole of Burkina Faso (Additional file 3). This is not uncommon when the reference set is constituted by large spatial extents such as whole countries, given that the degree of ecological specialization is generally directly proportional to the diversity of conditions accounted for by the reference set.

The two EGVs having by far the greatest impact upon the marginality factor of all the three taxa were distance from populated places and distance from roads (the topographic POPPL and ROAD predictors; cf. Table 1 and Additional files 4, 5 and 6). As these variables are related to the density of the human population and of roads, hence to the impact of anthropogenic modifications on the environment, this result is perhaps not surprising considering the high degree of anthropophily (*sensu *Besansky *et al*. [74]) generally expressed by *An. gambiae *and *An. arabiensis *in West Africa. Moreover, during the rainy season, unpaved roads are favourable to the occurrence of suitable larval breeding sites, and may favour the dispersal of mosquitoes by passive transport. Similarly, the EGV with the third highest positive load on the marginality factor for all species/forms was the frequency in each spatial unit of land exploited for farming annual crops (land use predictor CROP, cf. Table 1 and Additional files 4, 5 and 6), which is again a variable related to the degree of land occupancy and exploitation by humans.

The remaining four categories of land use differentiated *An. arabiensis *and the M form from the S form (Additional files 4, 5 and 6). Somewhat surprisingly, climatic EGVs were comparatively less influential than other classes of predictors. Only in the case of the S form did three out of four climatic EGVs have moderate coefficients on the marginality factor: this taxon preferred habitats with higher than average rainfall, and lower than average solar radiation and evapotranspiration, in agreement with its higher prevalence in the more humid savannas of the south. Conversely, *An. arabiensis *was positively associated with higher mean annual temperatures, consistent with its higher prevalence in the more arid savannas of the north. Only the S form was slightly associated with higher locations (EGV ALT), although it must be noted that the altitude range in Burkina Faso is limited, and this association probably resulted mostly from a correlation between the S southern distribution and the geographical localization of more mountainous regions in Burkina Faso (Figure 1).

The first specialization factor (Factor 2) of the ENFA, expresses the degree of tolerance of a species to occur in regions departing from the optimal habitat. The higher the factor load (in absolute value), the lower the ability of a species to cope with less-than-optimal environmental conditions. The minimum distance from roads and the frequency of croplands (ROAD and CROP predictors) had the highest coefficients for all three species/forms. This result, considered in the context of the disappearance of the POPPL predictor from this set, suggests that the impact of the human population on the ecological niche of these taxa is expressed mainly on rural habitats, presumably through the action of occupation and clearance of the savanna for agricultural purposes. In several instances, the specialization coefficient was moderately high for an environmental predictor of weak marginality (e.g. OPEN and FARM for *An. arabiensis*, RAIN and SUN for the M form, and SHRUB for the S form). This result is interpreted as the lack of tolerance of the focal species for the more extreme conditions expressed by that EGV, even if the optimum of the species coincides with the distribution of the EGV across the whole study area. In other instances the specialization coefficient was relatively high for EGVs of moderate marginality (in absolute terms). This was the case for the environmental predictors SHRUB for *An. arabiensis*, FARM and SHRUB for the M form, and RAIN and FARM for the S form. In this case, both marginality and specialization act in the same direction of preference by the focal species for more specific conditions concerning that EGV.

### Fundamental *vs*. realized ecological niche

**Figure 3:**
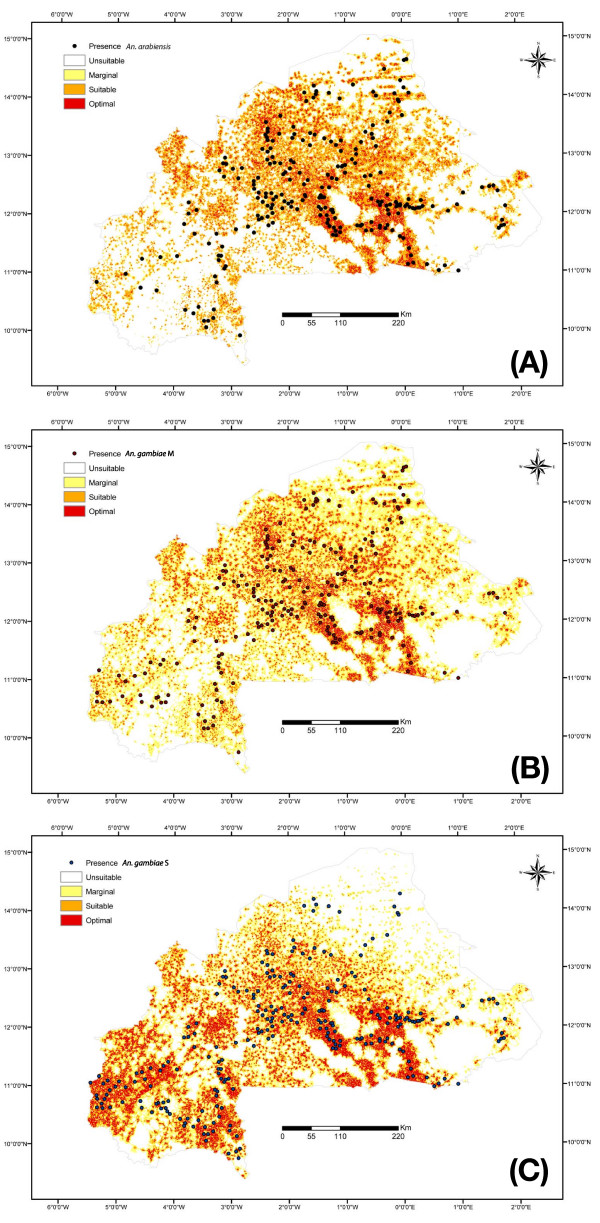
**Maps of the Habitat Suitability Index for members of the *Anopheles gambiae *complex in Burkina Faso**. Habitat suitability maps derived from the Ecological Niche Factor Analyses of (A) *An. arabiensis*; (B) *An. gambiae *molecular form M; and (C) *An. gambiae *molecular form S.

According to the broken stick rule, the Habitat Suitability (HS) maps exploited in all instances the first two factors of the ENFA, which explained 76–77% of the available information. The distribution of habitat quality across Burkina Faso presented some commonalities between the three taxa (Figure 3). Specifically, the region defined by the upper and medium basin of the Nakambé River was of high average quality (optimal to suitable) for all the three forms/species. This is an area of high population density and intensive farming (vegetables, millet, sorghum, cotton, and maize), characterized by numerous irrigation schemes (damming of small artificial lakes for village-scale farming, the large hydroelectric projects of the Kompienga and Bagré lakes and associated extensive rice cultivation schemes). The other commonality between the three taxa was the unsuitability of large, mostly uninhabited, areas occupied by national parks, game reserves, and forestry management projects.

On the other hand, a major difference between the taxa was the relative amount of suitable or optimal habitat available across Burkina Faso (Table 3): considerably more for both *An. arabiensis *and the S form (30–34% of the total surface) compared to the M form (22%), for which most habitat was of marginal quality (36%). In other words, the environment in Burkina Faso provided conditions that were mostly good to very good or totally unsuitable to *An. arabiensis *and the S form, whereas the same conditions were mostly marginal or unsuitable for the M form.

**Table 3:** Abundance of Habitat Suitability classes across Burkina Faso

Taxon	Frequency	Habitat Suitability			
		
		Unsuitable	Marginal	Suitable	Optimal
*An. arabiensis*					
	≥ 50%	22%	15%	47%	16%
	<50%	57%	13%	24%	6%
	*Overall*	53%	13%	27%	7%
					
*An. gambiae *M					
	≥ 50%	28%	42%	20%	10%
	<50%	50%	33%	11%	6%
	*Overall*	42%	36%	15%	7%
					
*An. gambiae *S					
	≥ 50%	39%	26%	19%	17%
	<50%	50%	26%	15%	9%
	*Overall*	44%	26%	17%	13%

Percent coverage of the study area by different bin classes of habitat suitability stratified by the interpolated relative frequency of members of the *An. gambiae *complex in Burkina Faso.

We categorized the study area into two classes according to the interpolated relative abundance of each taxon (≥50%, and ≤50%). For each taxon and abundance class, we assessed average habitat quality (unsuitable, marginal, suitable, optimal; Table 3).

Under the assumptions of equilibrium dynamics and in the absence of interspecific interactions, it is expected that the quality of the habitat as predicted by the fundamental ecological niche requirements should be positively correlated with the realized distribution and abundance of each taxon [75]. Theory also predicts that departures from this pattern – a signature of transient dynamics and/or interference among the taxa – should be characterized by the most recent (derived) taxon occupying habitat of more marginal quality than the ancestral taxa [44]. The predicted association between habitat quality and abundance matched more in the case of *An. arabiensis *and the S form, and less for the M form. The latter remained overly represented in habitat of marginal quality regardless of abundance status, whereas the former taxa were more abundant in habitat of overall higher quality (that is, optimal to suitable; Table 3).

The measures of model performance and robustness (respectively, Mean and SD in Table 4) for the HS maps indicated that the models were reasonably accurate for all the three forms/species, but were not particularly robust. This is a general feature of species distribution models of focal species having a widespread distribution with large marginality and restricted tolerance, as in our case, for purely methodological reasons [76]. Our main purpose, however, was not to predict with any degree of accuracy the occurrence of the focal species, but rather to infer gross patterns of habitat quality and distribution, and to rank the impact of environmental predictors on these. These patterns are less likely to have been strongly affected by the quality and robustness of the model predictions.

**Table 4:** Evaluation statistics for habitat suitability models

Taxon	Absolute Validation Index	Contrast Validation Index	Continuous Boyce Index
*An. arabiensis*			
Mean	0.55	0.34	0.45
SD	0.16	0.15	0.42
			
*An. gambiae *M			
Mean	0.53	0.34	0.55
SD	0.14	0.15	0.34
			
*An. gambiae *S			
Mean	0.51	0.33	0.61
SD	0.12	0.13	0.36

Model evaluation statistics for the habitat suitability maps shown in Figure 3, computed with 10-fold cross-validation. Larger values of the mean indicate greater accuracy. Smaller values of the standard deviation (SD) indicate greater robustness in model predictions.

### Ecological niche breadth and habitat overlap

The analysis of factor loads over the first discriminant factor isolated the main eco-geographical variables differentiating each pair of taxa. As shown in Table 5, different combinations of variables distinguished the environmental envelope characteristic of each taxon. However, the frequency distribution of the cell scores showed that in all cases the extent of overlap was substantial, indicating that the fundamental environmental envelope described by the selected EGVs did not differ markedly among the members of the complex (additional file 7).

**Table 5:** Eco-geographical variables discriminating the most the environmental envelope of different *An. gambiae s.l*. taxa in Burkina Faso

**EGV**	*An. gambiae *M *vs. An. arabiensis*	*An. gambiae *S *vs. An. arabiensis*	*An. gambiae *M *vs. An. gambiae *S
Climate			
RAIN	0.068	0.177	0.070
SUN	**-0.622**	**-0.513**	*0.294*
EVAPO	-0.109	**-0.215**	*0.462*
TEMP	**-0.262**	-0.046	-0.048
Land Use			
OPEN	0.066	**-0.328**	*0.509*
CROP	**-0.249**	-0.082	-0.042
FARM	*0.244*	0.078	**-0.275**
SHRUB	0.030	*0.254*	**-0.392**
FOREST	*0.413*	*0.312*	-0.061
Topography			
POPPL	-0.143	-0.023	-0.064
ROAD	-0.040	0.085	-0.003
HYDRO	-0.049	-0.112	0.000
ALT	*0.370*	*0.580*	**-0.333**
SLOPE	**-0.261**	-0.016	-0.149
ASPECT	0.033	-0.135	*0.248*
Eigenvalue	24.0	82.5	39.0
Explained variance	40%	57%	43%

Coefficients of the first discriminant factor between the habitat characteristics of pairs of *An. gambiae s.l*. taxa. Positive values (≥0.2 in italics) indicate variables that favour the first species of the pair, negative values (≤ -0.2 in bold) those that are favourable to the second species of the pair.

To quantify the degree of niche overlap we calculated three indices [64]: Levin's standardized niche breadth B*, Hurlbert niche overlap *L*, and Lloyd's directional interspecific crowding of species *y *on species *x*, Z_*x*(*y*)_. These measures are related to the probability that individuals of a species will encounter individuals of the other species. To assess the extent of ecological niche similarity and overlap between the fundamental *vs*. realized ecological niche of the three taxa, we calculated the indices either over the environmental envelope that maximally discriminated each pair of taxa (discriminant analysis, cf. Table 5 and additional file 7), or across the set of sampled locations (Table 6).

**Table 6:** Measures of ecological niche breadth and overlap

**Species pair**	**Niche Breadth**^†^		**Niche Overlap (L)**^§^		**Directional Overlap Z**_x(y)_*	
			
	**DA**	**SL**	**DA**	**SL**	**DA**	**SL**
*(1) An. arabiensis*	0.23	0.53	3.76	1.10	18.2	4.86
*(2) An. gambiae M*	0.27				17.7	5.65
						
*(1) An. gambiae S*	0.34	0.49	3.03	0.52	15.4	2.69
*(2) An. arabiensis*	0.27				14.7	3.55
						
*(1) An. gambiae M*	0.42	0.35	2.26	0.37	11.5	2.50
*(2) An. gambiae S*	0.40				10.7	1.63

† Levins' standardized niche breadth index B*; Eq. (28) in [64]

§ Lloyd's interspecies patchiness index *I*; Eq. (12) in [64]

* Loyd's asymmetric interspecific crowding index *Z*_*x*(*y*)_; Eq. (14) in [64]

Indices of ecological niche breadth, and overlap calculated either over the first discriminant axis segregating Species 1 from Species 2 of a pair (under columns "DA"), or across the sampled locations (under columns "SL"). The first approach provides measures of ecological segregation along the fundamental environmental envelope that discriminate most among the taxa (cf. additional file 7, and Table 5), whereas the second approach provides estimates of the realized degree of segregation between locations. We use the more general notation given by Hurlbert [64] of indices accounting for differences in availability of different resource states. The notation for the particular case of equally abundant resource states is provided as a footnote in the table.

*Anopheles arabiensis *had marginally lower indices of niche breadth than the two molecular forms, and the M form had marginally higher values than the S form (Table 6, under columns "DA"). The lowest values of Lloyd's asymmetric index were those between the two molecular forms, indicating that they segregated the most; an almost two-fold greater value was obtained for the degree of niche overlap between *An. arabiensis *and the M form, indicating that they segregated the least. The overlap was fairly symmetrical in all cases. The same conclusion was obtained by the Lloyd's interspecies patchiness index *I*: the two molecular forms had the lowest index (*L *= 2.26), *An. arabiensis vs. An. gambiae *M the highest (*L *= 3.76).

The estimates of niche breadth and habitat overlap obtained from the distribution and abundance of the three forms/species in the sample locations conformed to the general pattern of the discriminant analysis, but differences among taxa and species pairs were much larger (Table 6, under columns "SL"). Perhaps even more importantly, the degree of niche overlap was lower than that assessed from the fundamental environmental envelope (Table 6, under columns "DA"). The index of niche overlap was least between the M and S forms and 3-fold higher between *An. gambiae *form M and *An. arabiensis*. Similarly, Lloyd's asymmetric indices of interspecific crowding were lowest in the case of the M and S molecular forms, and 3–4-fold higher in the case of *An. arabiensis *and the M form. A comparison of these same indices across columns "DA" and "SL" in Table 6 reveals that the indices of niche overlap were 3–6 times greater when calculated along the environmental gradient that maximally discriminates among pairs of taxa. In other words, the realized habitat segregation of the taxa was more pronounced than that expected from the degree of habitat suitability across the study area.

Overall, these results indicate that the M form of *An. gambiae*, despite a potential niche breadth of similar magnitude as the other two taxa, which overlapped extensively with their fundamental ecological niche, realized a niche of lower breadth than *An. arabiensis *and *An. gambiae *form S, and diverged most from the latter taxon.

The process of ecological divergence between molecular forms has shifted the niche of the M form towards that of *An. arabiensis*, thereby inducing a higher niche overlap in the case of these two species.

### Chromosomal polymorphism and population structure

**Figure 4:**
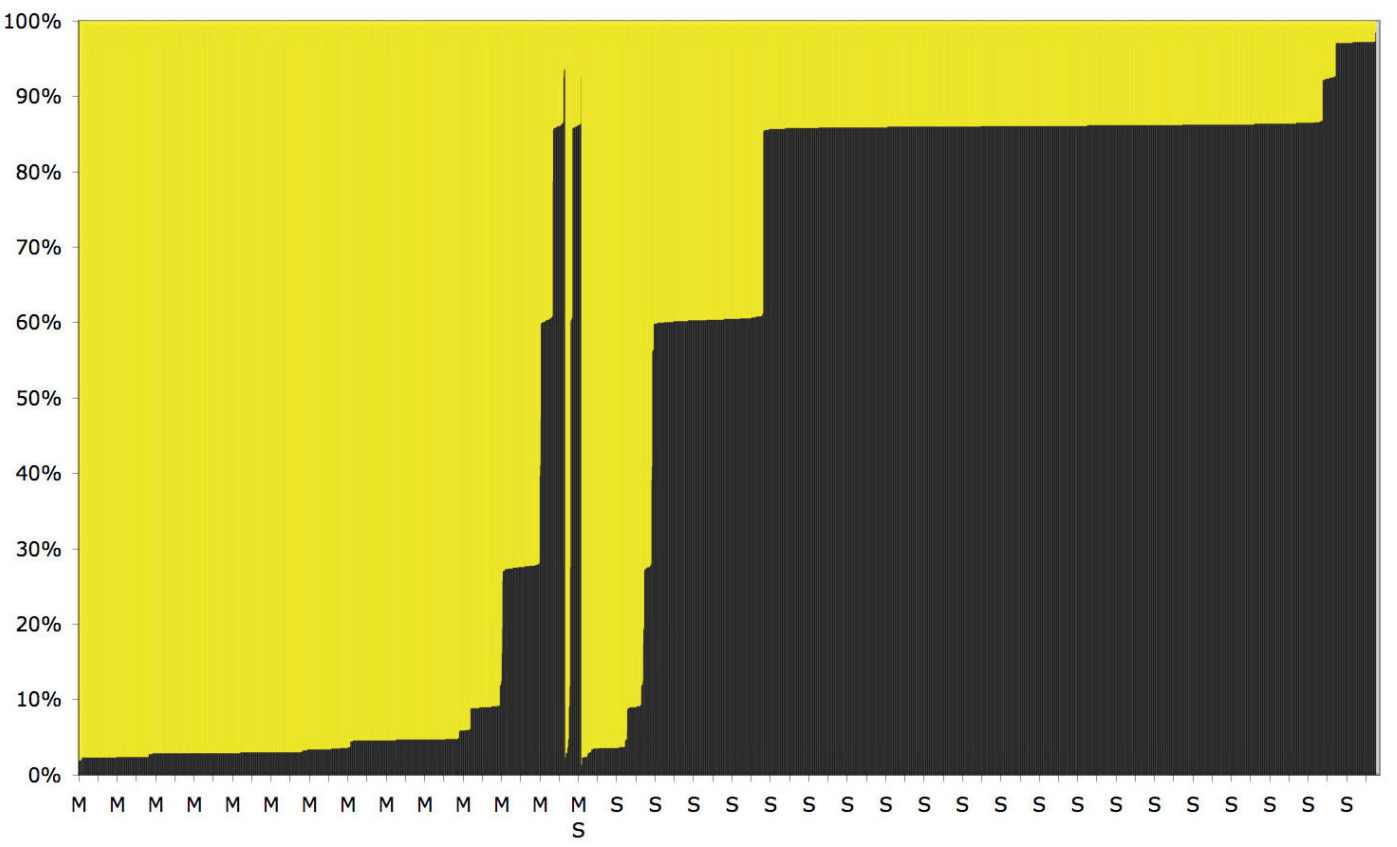
**Assignment of *An. gambiae s.s*. karyotypes by multilocus genetic clustering**. Results of the STRUCTURE analysis assuming *K *= 2 clusters (see additional file 8). The plot shows the probability that each of the 3,377 karyotyped mosquitoes, represented by a single bar along the abscissa, belongs to Cluster 1 (black bars). The corresponding probability value for Cluster 2 is the complement to 100% (yellow bars). The individual bars appear as solid colour because they are tightly spaced. Individual mosquitoes are ordered along the abscissa according to molecular form status (M, MS 'hybrids', S), and then by increasing probability of belonging to Cluster 1.

The chromosomal analysis was based on 3,377 *An. gambiae s.s*. that could be scored for all inversions. Table 7 presents the allocation of karyotypes by molecular form, by chromosomal form [31], and by genetic clusters of karyotypes assigned by STRUCTURE. STRUCTURE identified two clusters as the most likely based on the chromosomal data (additional file 8). Although most S karyotypes were assigned to Cluster 1 and M karyotypes to Cluster 2, the two clusters did not perfectly coincide with molecular form status (Figure 4 and Table 7). About 9% of S individuals were assigned to Cluster 2, and 5% of M-individuals to Cluster 1. As expected, karyotypes of the MS hybrids were much more widely shared between clusters (69% in Cluster 1 and 31% in Cluster 2 – Table 7). The chromosomal forms definition does not allow the unambiguous assignment of all possible karyotypes, either because of technical difficulties with scoring inversion haplotypes (the phase of variants on each chromatid), or because they are by definition 'recombinants' or otherwise segregating in multiple chromosomal forms (e.g. the 'FOREST' karyotypes that are shared between MOPTI and SAVANNA). These ambiguous karyotypes accounted for 13.5% of the samples across our study area. Thus, while some correspondence between molecular and chromosomal forms of *An. gambiae *exists in Burkina Faso as reported in [77], only 71.5% and 91.0% of all M and S specimens could be assigned to the chromosomal forms MOPTI and SAVANNA, respectively.

**Table 7:** Classification of karyotypes recorded from populations of *An. gambiae s.s*. across Burkina Faso

Karyotype	Molecular Form						Total	Chromosomal Form
			
	M		MS		S			
					
	Cluster		Cluster		Cluster			
	1	2	1	2	1	2		
00000	4	97	0	1	0	19	121	FOREST
00010	0	30	0	0	0	0	30	

00001	0	64	0	0	0	5	69	MOPTI
00002	0	7	0	0	0	0	7	
01100	0	284	0	2	0	5	291	
02200	0	399	0	4	0	9	412	
01101*	0	151	0	1	0	10	162	

01110	0	24	0	0	0	5	29	Recombinants and/or hybrids MOPTI/SAVANNA
01001	0	23	0	0	0	1	24	
01002	0	3	0	0	0	0	3	
02001	0	3	0	1	0	3	7	
02100	0	77	0	2	0	37	116	
02110	0	6	0	2	0	79	87	
02101**	0	26	0	0	0	5	31	

01000	32	0	6	0	319	0	357	SAVANNA
01010	0	7	0	0	0	0	7	
02000	27	0	23	0	1562	0	1612	
02010	1	1	0	0	0	1	3	
02202	0	0	0	0	0	1	1	

12000	0	0	0	0	4	0	4	Putative, rare SAVANNA karyotypes
11101	0	0	0	0	0	1	1	
12101	0	0	0	0	0	1	1	
11000	0	0	0	0	1	0	1	
12110	0	0	0	0	0	1	1	

Total	64	1202	29	13	1886	183	3377	

* depending on linkage this karyotype can be assigned to either SAVANNA or MOPTI

** depending on linkage this karyotype can belong either to SAVANNA or be considered as a hybrid MOPTI/SAVANNA

Assignment of karyotypes observed in Burkina Faso according to three classification criteria: the recorded rDNA IGS state (Molecular Form), the multi-locus genetic clustering approach implemented in the software STRUCTURE, without prior assignment to a given population (Clusters 1 and 2), and the chromosomal form status according to [31]. The non-Linnean nomenclature FOREST, MOPTI, and SAVANNA defines groups of karyotypes (i.e. chromosomal forms) restoring Hardy-Weinberg equilibrium of chromosomal inversion markers in field populations of *An. gambiae s.s*., with particular reference to Mali (for more details, see [31]). The table presents the frequency of observed karyotypes falling in each category.

It is interesting to note that the classification of karyotypes into chromosomal forms was originally proposed to restore genetic disequilibria observed in natural populations of *An. gambiae*. The original definition was mainly based on extensive sampling in Mali [31]. The same objective, by a different approach, is also the basis of the STRUCTURE algorithms. However, the two karyotype clusters identified by STRUCTURE from Burkina Faso samples are not coincident with the chromosomal form definitions (Table 7). Contrasting levels of chromosomal polymorphism distinguish the two clusters: Cluster 1 is characterized solely by the presence of the 2R*b*/+ 2L*a*/+ polymorphism, as encountered in *An. gambiae *populations across much of Africa, including east of the Great Rift Valley. Conversely, Cluster 2 is characterized by all other inversion arrangements (2R*c*, 2R*d*, 2R*u*) in combination with the 2R*b*.

If we accept the correspondence Cluster 1/S form and Cluster 2/M form, then "mismatches" between molecular form and cluster assignment by STRUCTURE would represent "admixed" individuals whose genotype is the result of shared ancestry or hybridization between the M and S forms. To explore this hypothesis, and to quantify the degree of admixture of different karyotypes, we repeated the STUCTURE analysis, this time after assignment of individual karyotypes to their empirically determined molecular form. In this case, STRUCTURE estimates the probability that each observed karyotype belongs to the population of origin, or whether it is the result of 'immigration' (in this case, shared ancestry or hybridization). The results of this analysis, shown in additional file 9, indicate that most karyotypes derived from M form specimens had a high probability (≥90%) of belonging to the M form. Karyotypes derived from the S form had a higher degree of admixture with M (probabilities ≤90%).

### Ecology of chromosomal variants

**Figure 5:**
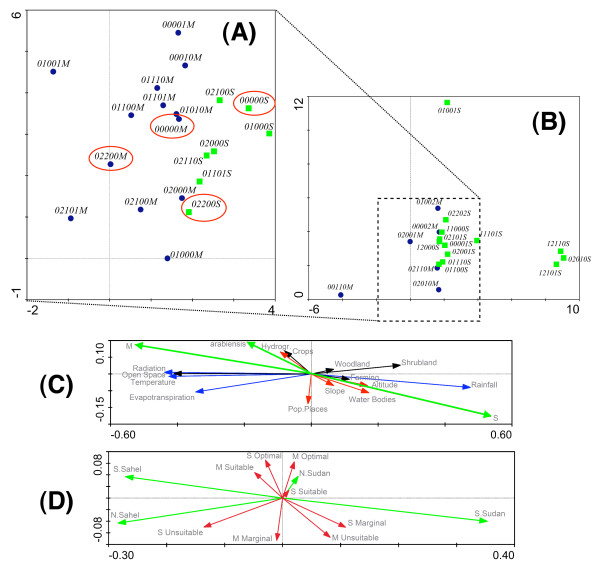
**Detrended Correspondence Analysis of *An. gambiae s.s*. karyotypes distribution across sampled locations by molecular form**. Distribution of karyotypes in ordination space, plotted over the first and second ordination axes. For visualization purposes, the diagram is split in four separate diagrams. Common karyotypes (weight in analysis >1%) are plotted in (A), rarer karyotypes in (B). Continuous eco-geographical variables (EGVs) are passively plotted in (C) and nominal variables in (D). Karyotypes of *An. gambiae *form M are designed by blue circles, those of the S form by green squares. In (C), climatic EGVs are symbolized by blue arrows, topographic variables by red arrows, land cover variables by black arrows and the molecular form relative abundance by green arrows. In (D), the vegetation classes are in green, and the habitat suitability classes are in red. Note that the scale is not the same across sub-diagrams. Circled karyotypes are those discussed in text.

The detrended correspondence analysis showed that the M and S form karyotypes segregated along the first ordination axis (Figure 5A and 5B), which accounted for *c*. 23% of the variance in the karyotype frequency data and *c*. 58% of the karyotype/environment correlation. The correlation between karyotypes and EGVs along this axis was very high (0.94). The contribution of the second axis was comparatively much lower: 7% of the karyotype frequency data and 6.5% of the karyotype/environment relation, with a correlation coefficient of 0.54. Similar values applied to the third and fourth axes. The first axis, therefore, captured most of the variability in the karyotype frequency distribution and the correlation of karyotypes with environmental conditions across the study area, although there remained a large portion of unaccounted variability that was spread over subsequent axes – each accounting for a relatively minor portion of the remaining unexplained variance. Despite the substantial number of EGVs, the amount of variance that the eco-geographical variables could account for was only moderate (32%).

To assist interpretation of the environmental gradients captured by the first and second ordination axes, continuous and nominal variables were plotted in ordination space (Figures 5C and 5D). The first ordination axis was highly correlated with the climatic variables and vegetation zones, suggesting that it defines the geographical (mainly latitudinal) cline associated with these EGVs: the right end of the axis was associated with higher values of rainfall and lower values of temperature, solar radiation, and evapotranspiration characteristic of the southern Sudan savanna vegetation; conversely higher values of temperature, solar radiation, and evapotranspiration and lower values of rainfall were associated with the two types of sahelian vegetation at the left end of the first ordination axis. The southern Sudan savanna region in Burkina Faso is associated with higher values of altitude due to a hilly region in the south-west (Figure 1), hence slope values are higher as well. Higher frequencies of woodland, shrubland, and areas of heterogeneous farming characterize the land cover of these same regions. Conversely, the Sahel is mostly characterized by areas devoid of vegetation (open spaces).

Interpretation of the second ordination axis is more difficult, but we suggest that it represents a gradient correlated with habitat quality. Unsuitable and marginal habitat classes for both M and S map in the lower part of the diagram, whereas suitable and optimal classes, again for both M and S, map in the upper part of the diagram. Distance from populated places, a variable that was highly influential in the ENFA, has a high negative correlation with the second ordination axis, that is, points falling in the lower part of the diagram are more distant from populated places, hence fall in habitat of lower quality according to the results of the ENFA for both M and S. This axis, therefore, would represent a gradient of overall habitat quality for *An*. *gambiae*, regardless of molecular form status.

Under this interpretation of the two main ordination axes, a model for the distribution of chromosomal variants in M and S can be proposed. The fact that M and S karyotypes strongly segregated along the first ordination axis is not surprising considering the observed pattern of geographical distribution of the two molecular forms across Burkina Faso (Figure 3). However, it is revealing to compare the response of the same karyotype segregating in different molecular forms. In the diagram, the relative position of a karyotype common to both molecular forms was consistent across forms overall. For example, the standard homokaryotype 00000M/S is found at the right end of both molecular form distributions; similarly, the 2R*bc/bc *karyotype 02200M/S is shifted to the left in both distributions (Figure 5A). Thus, the 00000M/S karyotypes were found in less 'arid' conditions in both M and S relative to the average conditions of aridity experienced by each form with respect to their geographical distribution, and the converse for 02200M/S karyotypes. In general, this suggests that although they are present in (and influenced by) different genetic backgrounds of the M or S form, each karyotype responded in a similar manner to environmental variables acting at a macrogeographic scale.

The distribution of karyotypes along the second environmental gradient, the one correlated with habitat quality, indicates that 'admixed' karyotypes within each molecular form occurred in habitat of overall less suitable quality. For example, the admixed karyotypes 02200S, 01100S, 01101S in S and 02000M, 02101M, 02100M, 01000M in M (see additional file 9 and Table 7) mapped at the lower end of the second ordination axis, whereas the 'typical' karyotypes were found higher up along this axis. To formally test this hypothesis we calculated the Spearman correlation coefficient between the scores on the second ordination axis of common karyotypes (total frequency >3) and the probability that the karyotype belonged to the population of origin (values in additional file 9). The correlation was positive and statistically significant (r_s _= 0.501, *n *= 31, *P *= 0.003), indicating that indeed, on average, more admixed karyotypes scored lower on the habitat quality gradient.

Several inferences can be drawn from these results: first, if alternative chromosomal arrangements were acting as selectively neutral alleles, one would expect similar karyotypes to map closely in ordination space along the first ordination axis. Contrary to this expectation, the same karyotypes in M and S were often distant along this axis, but not in a random order, consistent with the hypothesis that chromosomal inversions in *An. gambiae *are not selectively neutral. Second, the adaptive role of inversions is not absolute, but instead is conditioned upon the genetic background of the molecular form. Third, under the assumption that 'typical' karyotypes occurred in habitat of better quality because they are better adapted to it, the fitness disadvantage of 'admixed' karyotypes in marginal habitats of poorer quality might be offset by reduced intra-specific competition from 'typical' karyotypes. In other words, the 'admixed' karyotypes are those that 'live at the edge' of the adaptive landscape of each taxon, thereby providing the raw material on which evolutionary forces operate in the ecological speciation process.

## Discussion

The ecotypification theory of speciation of anopheline mosquitoes postulated by Coluzzi [8,78] is a specialized case of ecological speciation taking into account their small chromosome number (2N = 6) as well as their "flush and crash" population dynamics. Like ecological speciation generally, it is based on the coordinated action of ecological and genetic mechanisms for the evolution of reproductive isolation in diverging populations. The principal tenets and steps of this theory are (i) the invasion of a new ecological niche by a peripheral population; (ii) the appearance in the peripheral isolate and establishment (by positive selection) of alleles conferring a fitness advantage in the novel environment, in the absence of gene flow from the core population; (iii) the maintenance of linkage between these alleles in the face of gene flow from the core population by a genetic mechanism that suppresses recombination, notably paracentric chromosomal inversions; (iv) the increase of genetic differentiation between the ecologically diverging populations by accumulation of allelic differences, including genes involved in reproductive isolation.

From an ecological standpoint, this theory presupposes several ecological mechanisms, of which the invasion of a new niche is amongst the most crucial. When a peripheral population invades a new habitat, it is unlikely that it will already have acquired all the traits necessary to cope optimally with the novel environment. At the early stages of colonization, its fundamental ecological niche will be similar to that of the core population, resulting in extensive niche overlap between these populations. The new habitat, therefore, will be of marginal quality with respect to the requirements of the fundamental ecological niche of the invading peripheral population [44]. The occupation of the new habitat by the peripheral population, and its progressive adaptation to it, however, will reduce the *realized *niche breadth of the peripheral population and the degree of *realized *niche overlap between the peripheral and core populations. A transient mismatch between the fundamental *vs*. realized ecological niche of the core and peripheral populations, and the occurrence of the peripheral population in habitat of lower quality, therefore, constitute a signature of recent niche expansion. Later on, as the process of adaptation by the peripheral population to the new conditions carries on, its fundamental ecological niche will progressively change to match the environmental envelope characterizing the new habitat, producing a niche shift and a reduction in the extent of niche overlap between the core and peripheral populations.

The ecological niche properties of the M molecular form of *An. gambiae *that we recorded in populations from Burkina Faso fit with this signature of niche expansion and ecological divergence from the S form. The fundamental ecological niche of the two taxa inferred under the hypervolume framework formalized by Hutchinson [59], was rather similar: both forms need specific environmental requirements, whose occurrence is greatly influenced by the presence and activity of humans, and niche overlap is extensive even over the environmental gradient where the two forms segregate the most.

On the other hand, the biogeographic pattern of occurrence and abundance showed that the degree of realized niche overlap between the two molecular forms of *An. gambiae s.s*. was proportionally less, in discordance with expectations from ecological speciation theory which predicts, in taxa of similar age, an association between the amount of ecological divergence and the degree of reproductive isolation [1,5]. According to this prediction, we should have observed a higher degree of habitat segregation between the taxa of older ancestry – *An. gambiae *and *An. arabiensis*, characterized by efficacious pre-zygotic and post-zygotic mechanisms [8,13], than between the younger molecular forms of *An. gambiae*, among which reproductive isolation is at an earlier stage of development [20,22].

Departure from expectation might result from a combination of several factors. First, the association between ecological divergence and reproductive isolation is weaker when post-zygotic isolation is the main isolating mechanism [5]; post-zygotic isolation is stronger between *An. gambiae *and *An. arabiensis *than between the molecular forms of *An. gambiae *[13,22]. Second, we have examined only a subset of abiotic resources at a macro-geographic scale, whereas other factors (e.g. predation, competition) acting at other geographical scales might account for a different pattern of ecological differentiation among the three taxa. Third, in the process of niche expansion, the M molecular form of *An. gambiae*, which is presumably the most recent, has invaded part of the adaptive landscape of *An. arabiensis*. If chromosomal arrangements shared between these two taxa [9,32] were independently selected, genetic similarity might have resulted in ecological convergence.

**Figure 6:**
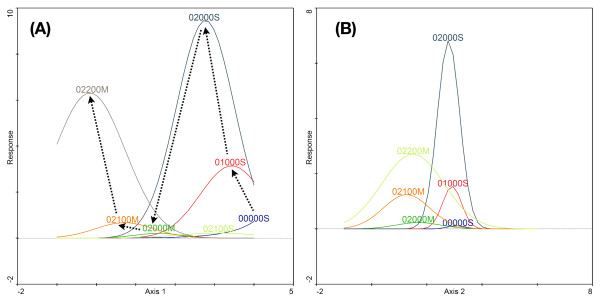
**Hypothetical evolutionary path leading to adaptive ecological divergence of some chromosome-2 variants of *Anopheles gambiae *molecular forms**. The figure shows second-order polynomial 'species' response curves [58,69] fitting the data set of karyotype frequencies (*P *< 0.01 in all cases). The response on the ordinate is a measure of relative abundance that is taken as a proxy of fitness. Axis 1 in (A) and Axis 2 in (B) on the abscissa are the same ordination axes as in Figure 5. They are interpreted to represent environmental gradients related to a major eco-geographical cline (Axis 1 – xeric conditions at higher latitudes on the left, mesic conditions at lower latitudes on the right), and to general habitat quality (Axis 2 – increasing habitat quality from left to right). The curves visualize the optimum response (the point on the abscissa falling at the maximum of the curve), and the degree of tolerance (the width of the curve around the optimum) of each karyotype along the environmental gradients. In (A), arrows point to a postulated sequence of chromosomal mutation and allele assortment events leading to a habitat shift from a monomorphic standard karyotype (00000S) to a typical Cluster 2/M karyotype (02200M) via typical Cluster 1/S (02000S) and then 'atypical' (02000M, 02100M) karyotypes. The letter "M" marks the appearance of ecologically adaptive genes in the independently segregating pericentromeric region of the X chromosome. The figure also shows that 02100S karyotypes share similar habitat optima and tolerance but lower fitness than 02000S karyotypes. In the face of competition with 02000S, therefore, 02100M and the 'atypical' 02000M karyotypes compete less against 02000S by occupying more marginal habitats, particularly on the habitat quality gradient (Axis 2 in B), compared to 02100S. In (B) it is apparent the greater degree of tolerance of M karyotypes, with optima shifted to habitat of overall lower quality relative to S. This evolutionary path is not exclusive and it is taken as an example for illustrative purposes: other paths involving different sets of karyotypes are also possible (not shown).

But what makes a population occupy habitat of marginal quality, and where does the process of niche expansion find its driving force? Our results suggest that competition between genetic variants is one of the possible mechanisms. Models of adaptive divergence across heterogeneous landscapes have demonstrated that competition can lead to the appearance of clusters of adaptive phenotypes at the extremes of an environmental cline [[79,80] and references therein], and there is experimental evidence that competition can lead to the expansion of niche width [81]. In our study area, the spatial distribution of chromosomal variants with respect to environmental variables and habitat quality was non-random. Instead, the same karyotypes were spatially distributed in a symmetric pattern in both molecular forms in response to gross environmental gradients of climate, vegetation, and land cover. This suggests two things. First, in agreement with a large literature [9,10,32,82], the polymorphic chromosomal inversions of *An. gambiae *constitute an adaptive system under balancing selection, playing a role in the ecological plasticity and adaptive potential of this species across a diversity of environments. Second, that particular karyotypes provide a broadly similar benefit to both M and S forms under the same environmental conditions. However, their particular ecological value appears to be conditioned on the different genetic backgrounds of the M and S forms. When karyotypes 'typical' of one form in Burkina Faso (for example, 02200 for M and 02000 for S) were carried by the other form (that is, 02200 in S and 02000 in M), they were observed in habitat of more marginal quality (Figure 5A and 6B), suggesting that these rarer 'atypical' karyotypes represent genotypes of overall lower fitness. Assuming that 'typical' karyotypes have superior fitness in suitable and optimal habitat, we propose that 'atypical' karyotypes are able to emerge from competition with 'typical' karyotypes only in marginal habitats, where the fitness advantage of the more successful 'typical' karyotypes becomes less (Figure 6). It is possible that 'atypical' karyotypes are those that initiate the process of niche expansion and shift. In the early steps of divergence, these karyotypes are probably maladapted to the marginal conditions they are obliged to live in to escape competition from the 'typical' karyotypes in the core of the adaptive landscape (Figure 6). However, the 'atypical' karyotypes may provide the raw material that evolution can build upon to initiate a process of ecological divergence. Were it not for an incomplete reproductive barrier between M and S, 'atypical' karyotypes (e.g. 02000M in Figure 6) would presumably go extinct once they have given rise to ecologically more successful karyotypes (e.g. 02200M in Figure 6), because of their lower overall fitness compared to other karyotypes along the entire environmental gradient. However, because of occasional hybridization between M and S, 'admixed' karyotypes still occur in natural field populations, occupying the same ecological role of their ancestral 'atypical' analogs.

**Figure 7:**
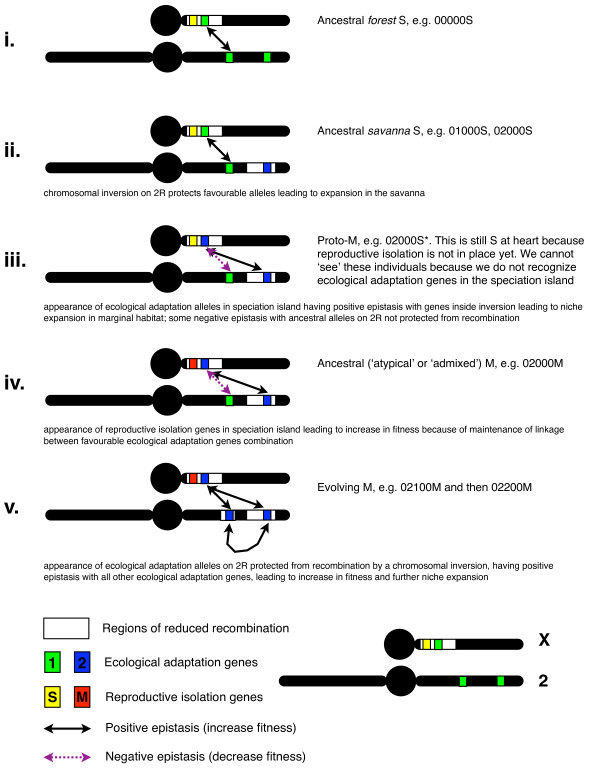
**Exemplifying evolutionary steps involved in the process of ecological adaptation and speciation of M and S**. Diagrammatic sequence of hypothetical genomic and chromosomal events on chromosome 2 and the X heterosome leading to ecological niche expansion and reproductive isolation in *An. gambiae *molecular forms following the evolutionary path exemplified in Figure 6.

How might these hypotheses relate to the ecotypification theory of speciation? The first observation is that chromosomal variants in *An. gambiae *populations are ubiquitous and emerge frequently. The analysis of the distribution and frequency of rare chromosomal inversions in this species agrees with a model of high chromosomal plasticity driven by the frequent emergence of chromosomal rearrangements on the 2R arm [83]. In general, most of these chromosomal variants do not capture enough adaptive alleles to go beyond the early phases of establishment, or they are otherwise selectively neutral and lost by drift. Based on the distribution of karyotypes in relation to the nature of habitat quality, we hypothesize that sometimes the rearrangements provide a selective advantage, but not enough to withstand the competition from genotypes already present in the core of the population adaptive landscape. These karyotypes are therefore confined to more marginal environments where they can initiate a process of niche expansion and niche shift. Yet there is a paradox, due to the observation that the same chromosomal inversion variants are shared between molecular forms of *An. gambiae*. This implies that while ecological adaptation genes should be found inside inversions on the 2R chromosomal arm, they are unlikely to contain the genes responsible for premating reproductive isolation between M and S. Indeed, the genome regions currently considered most likely to contain genes for reproductive isolation are highly diverged pericentromeric regions of chromosome X and 2L, termed "speciation islands" [28,84]. The supposition is that paracentric chromosomal inversions are not the sole mechanism by which ecotypification and speciation can take place. All genetic mechanisms by which recombination is suppressed, thereby providing a means to protect the linkage of favourable allele combinations, can play a role in this process – including suppressed recombination regions near centromeres [85]. Thus, it can be proposed that when ecologically favourable mutations in the pericentromeric "speciation islands" started to appear, the processes of ecological divergence and speciation arrived at a turning point. At this stage, the appearance of alleles controlling reproductive isolation could protect the linkage of the unlinked chromosomal regions (inversions and pericentromeric regions) harbouring ecologically-significant allele combinations. Another mechanism that could potentially foster this process would be any kind of selection against hybridization of the diverging ecotypes, such as e.g. reinforcement in the 'broad sense' [86]. Figure 7 schematically summarizes the postulated evolutionary steps of the proposed pattern of ecological and genetic diversification of M and S using the path of Figure 6 as a model, showing the dynamic interaction between ecological adaptation genes on different regions of the genome. It must be stressed that this specific path is only one possible realization that is taken to exemplify the role of 'typical' and 'atypical' karyotypes in the process of ecological niche expansion and the development of reproductive isolation between M and S. It is largely premature to conclude that this evolutionary path was precisely that followed during M and S speciation; we use it here only as a general model that we can use to visualize formally the logic of our argument and generate testable predictions against which future observations can be compared. A summary of the main results, hypotheses, and conclusions inferred from this study is presented in Table 8.

**Table 8:** Summary of main results, hypotheses, and conclusions

**Process**	**Patterns in favour**	**Patterns against or untested postulates**
Ecological divergence of molecular forms	Biogeographic differences in distribution; indices of co-occurrence and niche overlap; differences in distribution of habitat suitability; segregation of karyotypes in separate clusters coincident with molecular form status on major eco-geographical gradient (first DCA axis)	
Competition among taxa	Patterns of co-occurrence associated to relative abundance; reversal in relative abundance not matching environmental steepness of environmental clines; similar fundamental ecological niche (competition for the same resources)	Shared keystone predator(s) apparent competition
Niche expansion of M form	Mismatch between fundamental and realized ecological niches; relative prevalence in lower quality habitat	Phylogenetic relationship among taxa
Ecological divergence of karyotypes	Segregation along environmental gradients	
Competition among karyotypes	Segregation along habitat quality gradient of 'typical' and 'atypical' karyotypes	Shared keystone predator(s) apparent competition
Adaptive role of chromosomal inversions	Non-random distribution of karyotypes along major eco-geographical gradient; concordant distribution of karyotypes *between *molecular forms	Phylogeographic relationship among taxa; presence of ecological adaptation genes inside inversions
Role of chromosomal inversions in reproductive isolation (lack of)	Chromosomal inversions do not fully segregate according to molecular form	Presence of reproductive isolation genes inside speciation island(s)
Epistasis	Ecological value of chromosomal inversions depends upon M/S background	Presence of ecological adaptation genes inside speciation island(s)
Reinforcement	Rarity of hybrids (despite significant hybridization and fertility and viability of hybrids*)	Fitness of hybrids; cryptic mate choice
Secondary contact after divergence in allopatry	Admixture of karyotypes in contact zone	Mismatch between fundamental and realized ecological niches

* inferred from previously published literature

The present pattern of distribution of the diverging evolutionarily significant units of *An. gambiae*, the molecular forms M and S, agrees with the view that biogeographic patterns during speciation are dynamic [4]. The two forms are currently evolving reproductive isolation, and the present data show that they are ecologically diverging. Such ongoing speciation is happening under clinal sympatry, yet the very ecological processes underlying the initial steps of divergence presumably involve the occupation of marginal habitats and may happen under allopatry of the genetic variants responsible for the niche shift.

## Conclusion

*Anopheles gambiae*, the most significant vector for human malaria, shows a remarkable capacity to thrive in a wide range of environmental conditions, provided humans are present. Genomic regions of suppressed recombination appear to have a leading role in the adaptation of this species to novel environments and in the emergence of reproductive isolation. Ongoing speciation involving the two molecular forms of *An. gambiae *is accompanied, and presumably driven, by a process of ecological divergence initiated by expansion of the original ecological niche of the ancestral S form. Paracentric inversions on chromosomal arm 2R capturing allele combinations of ecological adaptive value were likely instrumental to the initiation of this process. The later appearance of ecologically significant genes in unlinked chromosomal regions such as the speciation islands is postulated for the evolution of reproductive isolation. Overall, these results highlight the phenomenal ecological and evolutionary flexibility of this mosquito, as well as the extraordinary complexity of the population structure and dynamics of this biological model – a lesson not only for students of speciation, but also for vector control.

## List of abbreviations

ASECNA: *Agence pour la Sécurité de la Navigation Aérienne en Afrique et à Madagascar *(Agency for the Safety of Aerial Navigation in Africa and Madagascar); AVI: Absolute Validation Index; BNDT: *Banque Nationale des Données Topographiques *(National Topographic Database); CVI: Contrast Validation Index; DCA: Detrended Correspondence Analysis; EGV: Eco-Geographical Variable; ENFA: Ecological Niche Factor Analysis; GIS: Geographical Information System; HSI: Habitat Suitability Index; IGB: *Institut Géographique du Burkina *(Burkina Faso Institute of Geography); IGS: Inter-Genic Spacer; LAT: Latitude; LONG: Longitude; MCMC: Markov Chain Monte Carlo; PCR: Polymerase Chain Reaction; rDNA: ribosomal Deoxy-Ribonucleic Acid; SDM: Species Distribution Modelling.

## Authors' contributions

The study was conceived by CC and NJB. CC, FS, and NJB devised the field protocol. WMG performed the field collections; WGM, MP and IHNB carried out the laboratory analyses under the supervision of NFS and CC. The geographical information system was conceived and implemented by CS and KO under the supervision of JMF. DA, CC, MP, and KO performed statistical analyses. CC, FS, NJB, and DF coordinated the project. CC wrote the article, which was critically revised by FS, NJB, and DF. All authors read and approved the manuscript.

## Supplementary Material

Additional file 1 **List of Sampled Locations**. Names and geographical coordinates of sampled villages, dates of collection, and sample size of collected and identified mosquitoes. Click here for file

Additional file 2 **Vegetation Classes in Burkina Faso**. Floristic associations defining the four main vegetation classes covering Burkina Faso that were used as supplementary EGVs in the DCA. Click here for file

Additional file 3 **Ecological Niche Factor Analysis statistics**. Marginality, Specialization, and Tolerance indices for the three taxa of the *An. gambiae s.l*. complex across Burkina Faso. Click here for file

Additional file 4 **Ecological Niche Factor Analysis coefficients for *An. arabiensis***. Factor loads of the 15 environmental predictors (EGVs). Factor 1 explains 100% of the marginality, the second and following factors explain increasing amounts of the specialization. Positive values of marginality indicate that *An. arabiensis *was found in locations with higher values than average for that EGV. Negative values for EGVs quantified as "minimum distance from" (POPPL, ROAD, HYDRO) indicate "preference to proximity". Higher specialization coefficients, regardless of sign, indicate that *Anopheles arabiensis *was found occupying a narrower range of values of the EGV than available in the reference set. For visualization purposes, values ≥0.2 are outlined in red, values ≤ -0.2 are outlined in blue. Click here for file

Additional file 5 **Ecological Niche Factor Analysis coefficients for *Anopheles gambiae *molecular form M**. Factor loads of the 15 environmental predictors (EGVs) for *An. gambiae s.s*. form M. Other symbols and explanations as for additional file 4. Click here for file

Additional file 6 **Ecological Niche Factor Analysis coefficients for *Anopheles gambiae *molecular form S**. Factor loads of the 15 environmental predictors (EGVs) for *An. gambiae s.s*. form S. Other symbols and explanations as for additional file 4. Click here for file

Additional file 7 **Discriminant analysis of habitat partitioning**. Relative frequency distribution of the cell scores occupied by forms/species of the *An. gambiae *complex in relation to the global distribution of all cells in the study area along the discriminant factor for which species pairs differed the most. Click here for file

Additional file 8 **Maximum likelihood estimates of population structure**. Values of Ln [Pr(*X*|*K*)], representing the probability of obtaining the observed genetic data *X *conditional on the presence of *K *populations (i.e. "clusters"), plotted against the number of genetic clusters *K *assumed in the population. Error bars are standard deviations of five replicate analyses for each value of *K *(some of the error bars are smaller than – hence hidden by – the circles representing the mean values they refer to). Click here for file

Additional file 9 **Admixture probabilities of different karyotypes**. Average probability (± SD) that a given karyotype belongs to the population of origin, in this case the recorded molecular form. Click here for file
