# Supplementary material for: Living at the edge: biogeographic patterns of habitat segregation conform to speciation by niche expansion in Anopheles gambiae
Source: BMC Ecol. 2009 May 21;9:16. doi: 10.1186/1472-6785-9-16 (PMC2702294; doi:10.1186/1472-6785-9-16)
Supplement: Additional file 2 — Vegetation Classes in Burkina Faso. Floristic associations defining the four main vegetation classes covering Burkina Faso that were used as supplementary EGVs in the DCA. [file 1472-6785-9-16-S2.pdf]

Vegetation Class	Vegetation sub-class (main floristic associations)
**North-Sahelian**	Swamps (Echinochloa stagnina)
	Thorn bush steppe (Acacia spp., Combretum spp.)
	Thorn bush steppe (Acacia tortilis ssp. raddiana)
	Thorn bush steppe (Combretum spp., Pterocarpus lucens)
	Thorn bush steppe (Acacia tortilis ssp. raddiana, A. laeta, Balanites aegyptiaca)
	Grassland steppe (Aristida spp.)
	Grassland or thorn bush steppe (Cenchrus biflorus, Combretum glutinosum, Balanites aegyptiaca)
**South-Sahelian**	Woodland savanna (Anogeisus leiocarpus)
	Thorn bush steppe (Combretum spp.)
	Thorn bush steppe (Combretum spp., Guiera senegalensis)
	Thorn bush steppe (Conretum nigricans, Guiera senegalensis) - Farming in valleys, Butyrospermum parkii and Acacia albida parks
	Thorn bush steppe with sparse trees (Butyrospermum parkii, Ziziphus mucronata)
	Mixed steppe and woodland savanna of valleys (Butyrospermum parkii, Lannea microcarpa) - Farming, B. parkii and A. albida parks
**North-Sudanese**	Mixed valley vegetation associated to farming -Butyrospermum parkii and Acacia albida parks
	Woodland savanna (Anogeisus leiocarpus) and flooded prairies of the Sourou river
	Woodland or open savanna (Anogeisus leiocarpus, Butyrospermum parkii, Laenna spp.)
	Woodland or open savanna (Combretum spp., Anogeisus leiocarpus, Butyrospermum parkii) - Farmland in valleys with B. parkii
	Woodland and forested savanna (Anogeisus leiocarpus)
**South-Sudanese**	Gallery forest (Berlinia grandifolia, Daniellia oliveri, Mitragyna inermis) and associated aquatic prairies
	Woodland and forested savanna (Burkea africana, Butyrospermum parkii, Pterocarpus erinaceus) - B. parkii and Parkia biglobosa parks
	Woodland and forested savanna and cleared forests (Isoberlinia doka, Burkea africana, Terminalia spp.) - B. parkii and Parkia biglobosa parks
	Woodland and forested savanna (Burkea africana, Crossopteryx febrifuga, Combretum spp.)
	Woodland and forested savanna (Butyrospermum parkii, Detarium microcarpum)
