# Supplementary material for: Living at the edge: biogeographic patterns of habitat segregation conform to speciation by niche expansion in Anopheles gambiae
Source: BMC Ecol. 2009 May 21;9:16. doi: 10.1186/1472-6785-9-16 (PMC2702294; doi:10.1186/1472-6785-9-16)
Supplement: Additional file 3 — Ecological Niche Factor Analysis statistics. Marginality, Specialization, and Tolerance indices for the three taxa of the An. gambiae s.l. complex across Burkina Faso. [file 1472-6785-9-16-S3.pdf]

**Index**	*An. arabiensis*	*An. gambiae* M	*An. gambiae* S
Marginality	1.02	1.02	1.03
Specialisation	1.56	1.57	1.53
Tolerance	0.64	0.64	0.65
