# Supplementary material for: Living at the edge: biogeographic patterns of habitat segregation conform to speciation by niche expansion in Anopheles gambiae
Source: BMC Ecol. 2009 May 21;9:16. doi: 10.1186/1472-6785-9-16 (PMC2702294; doi:10.1186/1472-6785-9-16)
Supplement: Additional file 4 — Ecological Niche Factor Analysis coefficients for An. arabiensis. Factor loads of the 15 environmental predictors (EGVs). Factor 1 explains 100% of the marginality, the second and following factors explain increasing amounts of the specialization. Positive values of marginality indicate that An. arabiensis was found in locations with higher values than average for that EGV. Negative values for EGVs quantified as "minimum distance from" (POPPL, ROAD, HYDRO) indicate "preference to proximity". Higher specialization coefficients, regardless of sign, indicate that Anopheles arabiensis was found occupying a narrower range of values of the EGV than available in the reference set. For visualization purposes, values ≥0.2 are outlined in red, values ≤ -0.2 are outlined in blue. [file 1472-6785-9-16-S4.pdf]

EGV	Factor														
	1	2	3	4	5	6	7	8	9	10	11	12	13	14	15
RAIN	-0.05	0.18	0.11	0.45	0.44	-0.36	0.17	0.72	-0.60	0.59	-0.40	-0.53	-0.55	0.62	-0.43
SUN	0.07	0.10	-0.42	0.30	0.27	-0.01	-0.10	0.33	-0.01	0.28	-0.79	0.26	0.17	0.68	-0.21
EVAPO	-0.01	0.06	0.09	-0.27	0.19	0.32	0.37	0.39	0.12	0.28	-0.10	-0.26	0.07	-0.17	-0.01
TEMP	0.13	-0.13	0.18	0.58	-0.36	-0.03	0.62	-0.07	-0.36	-0.11	0.24	-0.42	-0.46	0.12	-0.14
OPEN	-0.06	0.24	0.23	-0.21	0.32	0.02	0.02	0.19	-0.46	0.04	0.21	0.17	-0.50	0.03	0.47
CROP	0.36	0.65	0.20	0.16	0.22	0.14	0.29	-0.11	-0.05	0.03	0.01	0.34	-0.11	-0.04	-0.03
FARM	0.07	0.36	-0.02	0.04	0.14	0.03	0.13	-0.01	0.16	-0.48	0.04	-0.19	0.11	0.09	0.19
SHRUB	-0.20	0.21	0.25	0.32	0.07	-0.24	0.51	0.10	-0.01	-0.04	0.00	0.36	0.04	-0.06	0.61
FOREST	-0.17	-0.01	0.42	0.28	0.06	0.66	-0.08	-0.14	0.14	0.14	-0.04	-0.04	0.03	0.02	0.08
POPPL	-0.76	-0.05	0.17	0.02	0.13	-0.16	0.13	-0.10	-0.01	-0.25	-0.03	0.12	-0.04	0.02	-0.29
ROAD	-0.43	0.53	-0.43	0.05	-0.32	0.34	-0.05	0.05	-0.02	0.22	0.05	-0.10	0.01	0.04	0.07
HYDRO	-0.05	-0.06	-0.01	-0.06	0.08	-0.29	0.12	-0.13	0.31	0.29	0.08	-0.14	-0.22	0.19	0.02
ALT	0.04	0.06	0.47	-0.19	-0.50	-0.03	0.18	-0.09	-0.14	0.00	-0.02	0.02	0.10	0.14	-0.10
SLOPE	0.03	0.06	-0.08	0.02	-0.06	-0.01	-0.07	-0.06	0.27	-0.16	-0.23	-0.02	-0.36	-0.14	0.03
ASPECT	0.07	-0.03	0.09	0.11	-0.06	0.14	-0.04	0.32	0.22	-0.09	0.18	0.21	0.01	0.10	-0.13
Expl. Specialization	41%	11%	7%	6%	5%	5%	4%	3%	3%	3%	3%	3%	2%	2%	1%
Cum. Expl. Spec.	41%	52%	59%	65%	70%	74%	78%	81%	84%	88%	90%	93%	95%	97%	100%
