# Supplementary material for: Living at the edge: biogeographic patterns of habitat segregation conform to speciation by niche expansion in Anopheles gambiae
Source: BMC Ecol. 2009 May 21;9:16. doi: 10.1186/1472-6785-9-16 (PMC2702294; doi:10.1186/1472-6785-9-16)
Supplement: Additional file 6 — Ecological Niche Factor Analysis coefficients for Anopheles gambiae molecular form S. Factor loads of the 15 environmental predictors (EGVs) for An. gambiae s.s. form S. Other symbols and explanations as for additional file 4. [file 1472-6785-9-16-S6.pdf]

EGV	Factor														
	1	2	3	4	5	6	7	8	9	10	11	12	13	14	15
RAIN	0.16	0.32	0.39	0.37	0.27	0.28	0.38	-0.27	-0.65	0.03	-0.33	0.26	0.76	-0.39	0.51
SUN	-0.14	0.08	0.11	-0.17	0.16	0.04	0.43	-0.20	-0.07	0.73	0.25	-0.55	0.58	-0.19	0.28
EVAPO	-0.13	-0.16	0.46	0.57	0.46	0.39	0.11	-0.13	0.02	0.03	-0.03	-0.00	-0.01	-0.09	-0.02
TEMP	-0.08	0.10	-0.29	0.29	0.42	-0.32	-0.51	0.30	-0.24	-0.54	-0.25	0.17	0.20	-0.00	0.23
OPEN	-0.19	0.07	0.54	-0.10	-0.14	-0.18	0.05	-0.41	-0.47	-0.25	-0.35	0.32	0.06	0.36	-0.40
CROP	0.28	0.63	0.36	0.11	0.14	-0.40	0.06	-0.09	-0.10	0.00	-0.11	-0.22	-0.07	0.29	0.09
FARM	0.15	0.31	0.29	-0.02	0.13	-0.21	-0.26	0.21	0.17	0.07	0.27	0.30	0.01	-0.13	-0.19
SHRUB	-0.08	0.33	0.03	0.45	0.21	-0.37	-0.22	-0.23	-0.23	0.10	0.08	-0.14	-0.01	0.42	-0.55
FOREST	-0.08	0.00	-0.11	-0.01	0.37	-0.31	0.43	0.02	0.08	-0.07	0.10	-0.02	-0.03	-0.15	-0.09
POPPL	-0.75	0.02	0.02	0.14	-0.16	-0.26	-0.06	0.08	-0.06	0.10	-0.12	0.10	-0.05	-0.22	0.25
ROAD	-0.42	0.51	0.05	-0.33	0.33	0.32	-0.05	-0.06	0.12	-0.13	0.07	-0.22	0.05	0.08	-0.07
HYDRO	-0.10	0.05	-0.01	0.25	-0.31	-0.09	0.09	-0.18	0.28	-0.20	0.08	0.04	0.15	0.05	0.02
ALT	0.16	0.01	-0.11	-0.01	0.04	-0.15	-0.26	-0.20	-0.05	-0.11	-0.14	-0.47	-0.02	-0.54	0.00
SLOPE	0.07	0.03	-0.05	-0.01	0.09	-0.07	0.05	0.06	0.31	0.13	-0.65	0.14	0.05	0.17	-0.02
ASPECT	0.05	-0.03	-0.07	-0.05	0.20	-0.02	-0.12	-0.65	-0.02	0.02	0.29	0.23	-0.04	0.04	0.17
Expl. Specialization	37%	16%	7%	6%	5%	4%	4%	4%	4%	3%	3%	2%	2%	2%	1%
Cum. Expl. Spec.	37%	53%	60%	66%	71%	76%	80%	83%	87%	90%	92%	95%	97%	99%	100%
