# Supplementary figures and images for: Living at the edge: biogeographic patterns of habitat segregation conform to speciation by niche expansion in Anopheles gambiae
Source: BMC Ecol. 2009 May 21;9:16. doi: 10.1186/1472-6785-9-16 (PMC2702294; doi:10.1186/1472-6785-9-16)

(A)

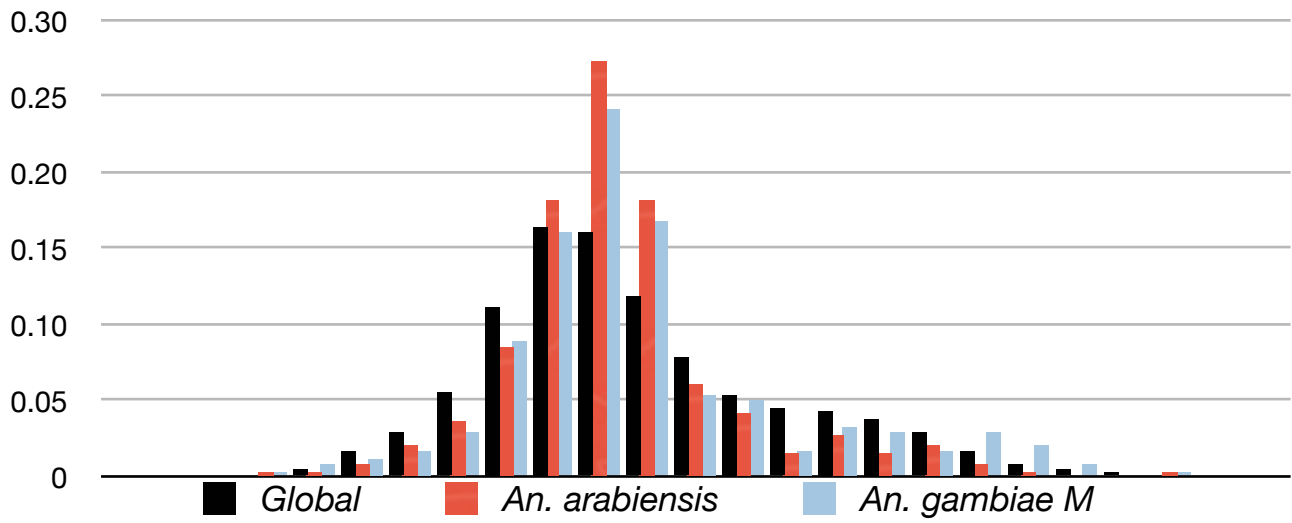

(B)

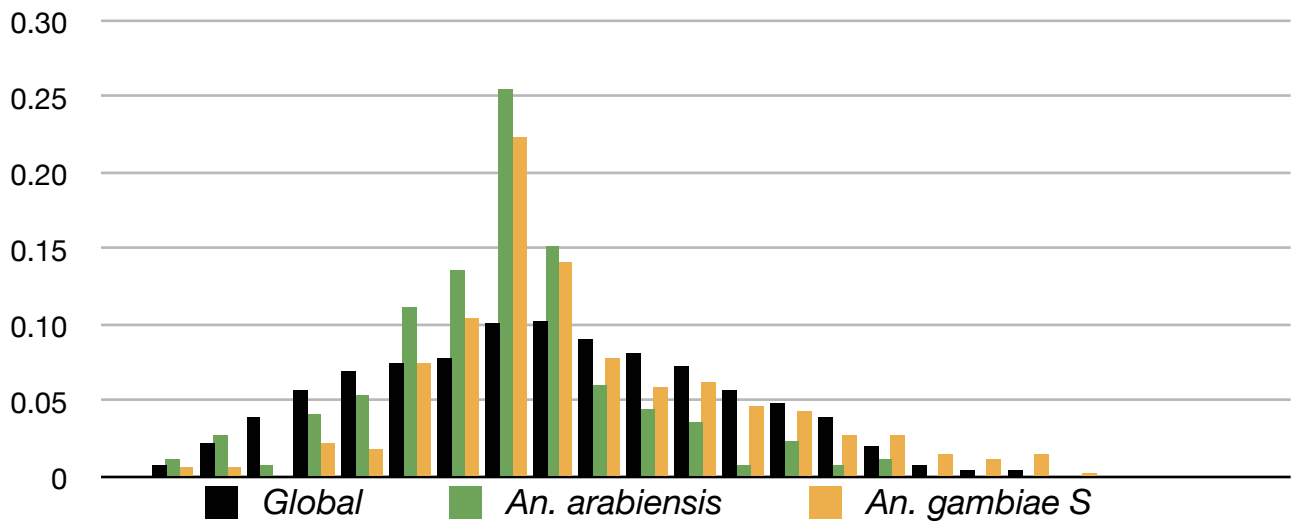

(C)

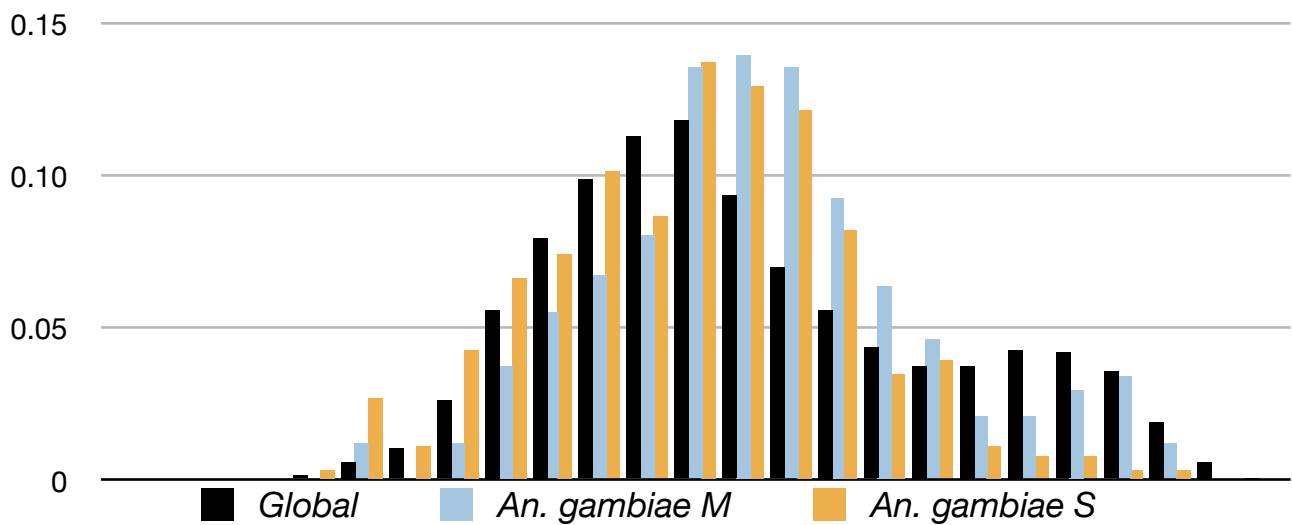

Supplement: Additional file 7 — Discriminant analysis of habitat partitioning. Relative frequency distribution of the cell scores occupied by forms/species of the An. gambiae complex in relation to the global distribution of all cells in the study area along the discriminant factor for which species pairs differed the most. [file 1472-6785-9-16-S7.pdf]

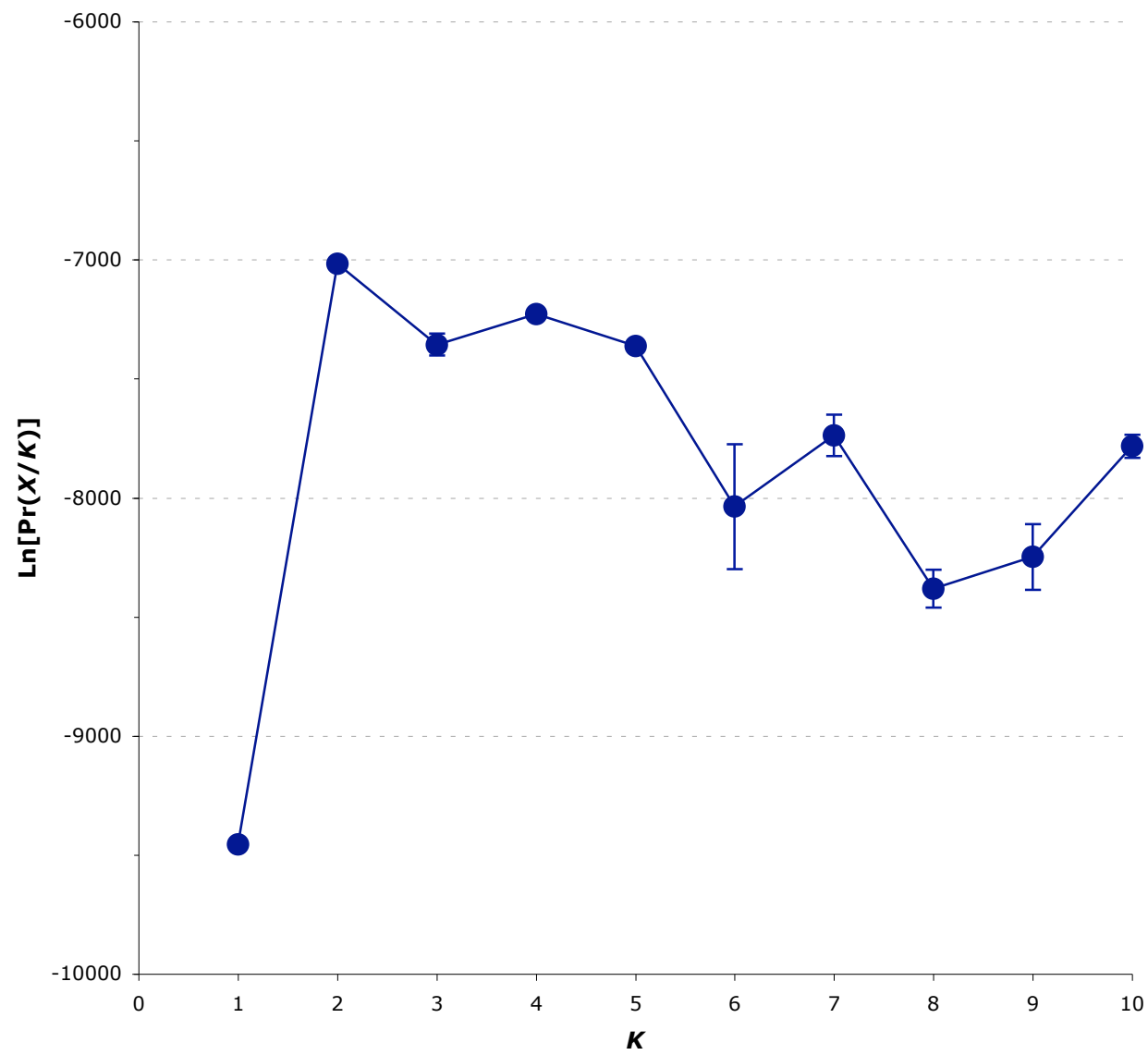

Supplement: Additional file 8 — Maximum likelihood estimates of population structure. Values of Ln [Pr(X|K)], representing the probability of obtaining the observed genetic data X conditional on the presence of K populations (i.e. "clusters"), plotted against the number of genetic clusters K assumed in the population. Error bars are standard deviations of five replicate analyses for each value of K (some of the error bars are smaller than – hence hidden by – the circles representing the mean values they refer to). [file 1472-6785-9-16-S8.pdf]
