# Supplementary material for: Living at the edge: biogeographic patterns of habitat segregation conform to speciation by niche expansion in Anopheles gambiae
Source: BMC Ecol. 2009 May 21;9:16. doi: 10.1186/1472-6785-9-16 (PMC2702294; doi:10.1186/1472-6785-9-16)
Supplement: Additional file 9 — Admixture probabilities of different karyotypes. Average probability (± SD) that a given karyotype belongs to the population of origin, in this case the recorded molecular form. [file 1472-6785-9-16-S9.pdf]

Karyotype	Taxon		
	M	MS	S
00000	0.974 ± 0.016	0.217 ± 0.000	0.798 ± 0.000
00001	0.984 ± 0.000	0.000 ± 0.000	0.016 ± 0.000
00002	0.991 ± 0.000	0.000 ± 0.000	0.000 ± 0.000
00010	0.981 ± 0.000	0.000 ± 0.000	0.000 ± 0.000
01000	0.788 ± 0.044	0.651 ± 0.058	0.929 ± 0.006
01001	0.875 ± 0.000	0.000 ± 0.000	0.078 ± 0.000
01002	0.947 ± 0.000	0.000 ± 0.000	0.000 ± 0.000
01010	0.839 ± 0.000	0.000 ± 0.000	0.000 ± 0.000
01100	0.980 ± 0.002	0.174 ± 0.000	0.478 ± 0.000
01101	0.986 ± 0.002	0.117 ± 0.000	0.004 ± 0.003
01110	0.983 ± 0.000	0.000 ± 0.000	0.316 ± 0.000
02000	0.339 ± 0.000	0.854 ± 0.001	0.984 ± 0.001
02001	0.786 ± 0.000	0.584 ± 0.000	0.272 ± 0.000
02010	0.339 ± 0.000	0.000 ± 0.000	0.978 ± 0.000
02100	0.810 ± 0.025	0.477 ± 0.000	0.727 ± 0.012
02101	0.871 ± 0.031	0.000 ± 0.000	0.018 ± 0.000
02110	0.848 ± 0.000	0.396 ± 0.000	0.637 ± 0.014
02200	0.983 ± 0.002	0.152 ± 0.000	0.183 ± 0.000
02202	0.000 ± 0.000	0.000 ± 0.000	0.000 ± 0.000
11000	0.000 ± 0.000	0.000 ± 0.000	0.948 ± 0.000
11101	0.000 ± 0.000	0.000 ± 0.000	0.020 ± 0.000
12000	0.000 ± 0.000	0.000 ± 0.000	0.987 ± 0.000
12101	0.000 ± 0.000	0.000 ± 0.000	0.043 ± 0.000
12110	0.000 ± 0.000	0.000 ± 0.000	0.725 ± 0.000
